# Delegated agency and moral responsibility in artificial intelligence

**DOI:** 10.3389/frai.2026.1800302

**Published:** 2026-04-15

**Authors:** Petar Radanliev

**Affiliations:** 1 Department of Computer Sciences, University of Oxford, Oxford, United Kingdom; 2 The Alan Turing Institute, British Library, London, United Kingdom

**Keywords:** delegated moral authority, artificial intelligence ethics, AI governance, moral responsibility in AI, autonomous AI systems, human-in-the-loop control, algorithmic accountability, responsibility gaps in AI

## Abstract

**Introduction:**

Artificial intelligence ethics is often framed as a response to unprecedented technical autonomy, with risks attributed to recent advances in machine learning and scale. This framing overlooks a recurring ethical structure: the delegation of moral authority to artificial agents. Ethical failures associated with AI are best understood as governance failures rooted in human design choices and accountability arrangements, even where opacity and limited control complicate responsibility attribution.

**Methods:**

A qualitative, interdisciplinary approach integrates historical–thematic analysis, comparative interpretation of technological artifacts, and visual–conceptual synthesis. Mythological figures (Talos, the Golem, Pygmalion), early mechanical automata, and foundational computational systems are analyzed as conceptual models of delegated artificial agency rather than technological precursors.

**Results:**

Across historical contexts, artificial agents exhibit consistent structural features: bounded autonomy, delegated authority, explicit override mechanisms, and dependence on human oversight. These features directly correspond to contemporary AI ethics concerns, including alignment failures, responsibility gaps, human-in-the-loop control, and system interruptibility.

**Discussion:**

The analysis establishes that ethical risk in AI arises from the displacement of human responsibility rather than from machine autonomy. By situating AI within a longer history of artificial agency, the study provides a normative framework that locates moral responsibility unambiguously in human actors and institutions, with direct implications for AI governance and accountability.

## Introduction

Artificial intelligence (AI) is commonly framed in contemporary discourse as a historically unprecedented source of moral risk. Concerns related to alignment failures, loss of human control, responsibility gaps, and existential threat are frequently attributed to recent advances in machine learning, system autonomy, and computational scale. Within this framing, ethical challenges are understood primarily as consequences of novel technical capabilities that exceed established modes of governance. While this perspective identifies genuine risks, it obscures a more fundamental issue: many ethical problems associated with AI arise not from technological novelty, but from long-standing human practices of delegating authority, responsibility, and decision-making to artificial agents.

Across cultures and historical periods, humans have repeatedly designed artifacts, mythological, mechanical, and computational, to act on their behalf, often in protective, supervisory, or decision-making roles. These artifacts have consistently embodied delegated human intentions while simultaneously generating ethical concern when their operation revealed design vulnerabilities, unclear accountability, or failures of oversight. From this vantage point, AI does not introduce a new category of moral threat; instead, it intensifies enduring governance problems associated with the externalization of moral authority into non-human systems.

Public debate often centers on whether AI systems might act independently of human values or intentions, potentially causing large-scale harm. A more precise ethical focus lies elsewhere. Harmful outcomes attributed to AI are better explained by human design choices, institutional incentives, and governance failures than by machine autonomy itself. The central ethical problem is therefore not whether AI systems possess moral agency, but how moral responsibility is allocated, constrained, and enforced when authority is delegated to artificial systems operating at speed, scale, and organizational distance.

Understanding this problem requires situating AI within a broader historical continuum of artificial agency. Conceptual models that predate modern computing nonetheless articulate structural features that remain ethically salient. The Greek myth of Talos depicts an automated guardian with bounded autonomy and a single point of failure. Jewish folklore presents the Golem as a conditionally obedient protector subject to explicit human override. Mechanical automata developed in Islamic and European contexts demonstrate early attempts to formalize delegated action through engineered constraint. Foundational computational artifacts associated with Babbage and Lovelace further illustrate how human intention, abstraction, and control become embedded in programmable systems. These cases are not technological precursors to AI, but analytical models that expose recurring features of delegated agency, including operational fragility, limited contextual judgement, and dependence on human oversight.

Narratives of anthropomorphism and affective projection further complicate the ethical landscape. The myth of Pygmalion and Galatea illustrates a persistent tendency to attribute moral and emotional qualities to artificial entities. This tendency remains ethically consequential in contemporary contexts, where conversational and generative AI systems simulate empathy, creativity, and intentionality. Such simulations encourage misattribution of moral agency, thereby weakening scrutiny of the human actors and institutions responsible for system design, deployment, and governance. This dynamic helps explain why responsibility gaps persist even in environments with formal technical safeguards.

A historically informed and conceptually grounded approach clarifies that existing ethical principles, such as accountability, oversight, and alignment, do not fail due to conceptual insufficiency, but due to systematic displacement of responsibility. Delegated moral authority, when combined with scale and abstraction, renders human accountability opaque unless explicitly constrained by institutional mechanisms. Ethical governance of AI therefore depends on maintaining clear lines of responsibility, enforceable oversight, and meaningful intervention capacity throughout the system lifecycle.

Situating AI within the longer history of artificial agency provides a framework for understanding why ethical failures recur despite advances in technical capability. The primary ethical challenge lies not in constraining machines as moral actors, but in preventing humans from relinquishing moral responsibility to the artifacts they design and deploy. Recognizing AI as a mirror of human moral agency establishes a more precise foundation for analyzing risk, responsibility, and governance in contemporary AI systems.

The study engages with a cross-disciplinary methodology, drawing upon mythological, philosophical, and technological narratives to trace the human fascination with artificial beings. From the mythic figure of Talos in Ancient Greece (Robertson, 1977) and the clay-based Golem of Jewish folklore (Winkler, 1980), to the mechanical automata of Al-Jazari (Kline, 2011) and Leonardo da Vinci’s Robot Lion, the history of AI is embedded within a much older cultural history of artificial agency. These historical artifacts reflect our desire for control and protection but also our persistent unease with the idea of machines imbued with power. There is also the other side of this historical connection with artificial intelligence, which is reflected in the myth of Pygmalion and Galatea (Rodin, 1889), in which a statue is brought to life by its creator’s love, invites us to consider artificial intelligence’s emotional and ethical implications. It is questioned whether future AI can incorporate human characteristics such as love and relationships into their programming.

The cases selected in this study are not exhaustive representations of artificial beings in cultural history. They are chosen because each isolates a distinct structural feature of delegated agency: Talos (bounded coercive mandate and vulnerability), the Golem (conditional activation and override), Leonardo’s automata (mechanical programmability), the Jaquet-Droz Writer (symbolic execution), and Pygmalion (anthropomorphic projection and the threshold of subjecthood). Other relevant figures, such as Frankenstein’s creature or Čapek’s R. U. R., foreground different themes, including industrial exploitation and creator responsibility, but operate within a later literary tradition shaped by modern technological anxiety. The present selection prioritizes structural clarity over chronological exhaustiveness.

### Scholarly contribution and argumentative positioning

This article advances a normative and conceptual argument rather than a systematic literature review. Its primary contribution is to reframe contemporary debates on artificial intelligence risk and moral agency by situating them within a long historical lineage of human-designed artificial protectors. Drawing on mythological, mechanical, and early computational artifacts, the paper argues that contemporary concerns regarding AI autonomy, alignment, and existential risk are best understood as recurring manifestations of a deeper and persistent pattern: the externalization of human moral responsibility into artifacts endowed with delegated agency.

The central claim advanced is that artificial intelligence does not introduce a fundamentally new category of moral threat. Instead, AI functions as a socio-technical mirror that amplifies pre-existing human tendencies toward control, protection, delegation, and abdication of responsibility. By analyzing figures such as Talos, the Golem, and Pygmalion alongside early programmable machines, the paper demonstrates that the ethical risks associated with AI stem less from machine autonomy than from human design choices, governance failures, and moral outsourcing.

This contribution complements contemporary AI ethics literature on alignment, responsibility gaps, and human–machine interaction by offering a historically grounded conceptual framework. Rather than proposing new ethical principles, the paper clarifies the conditions under which ethical failures recur across technological epochs, thereby supporting more reflexive and historically informed approaches to AI governance.

**Figure 1:**
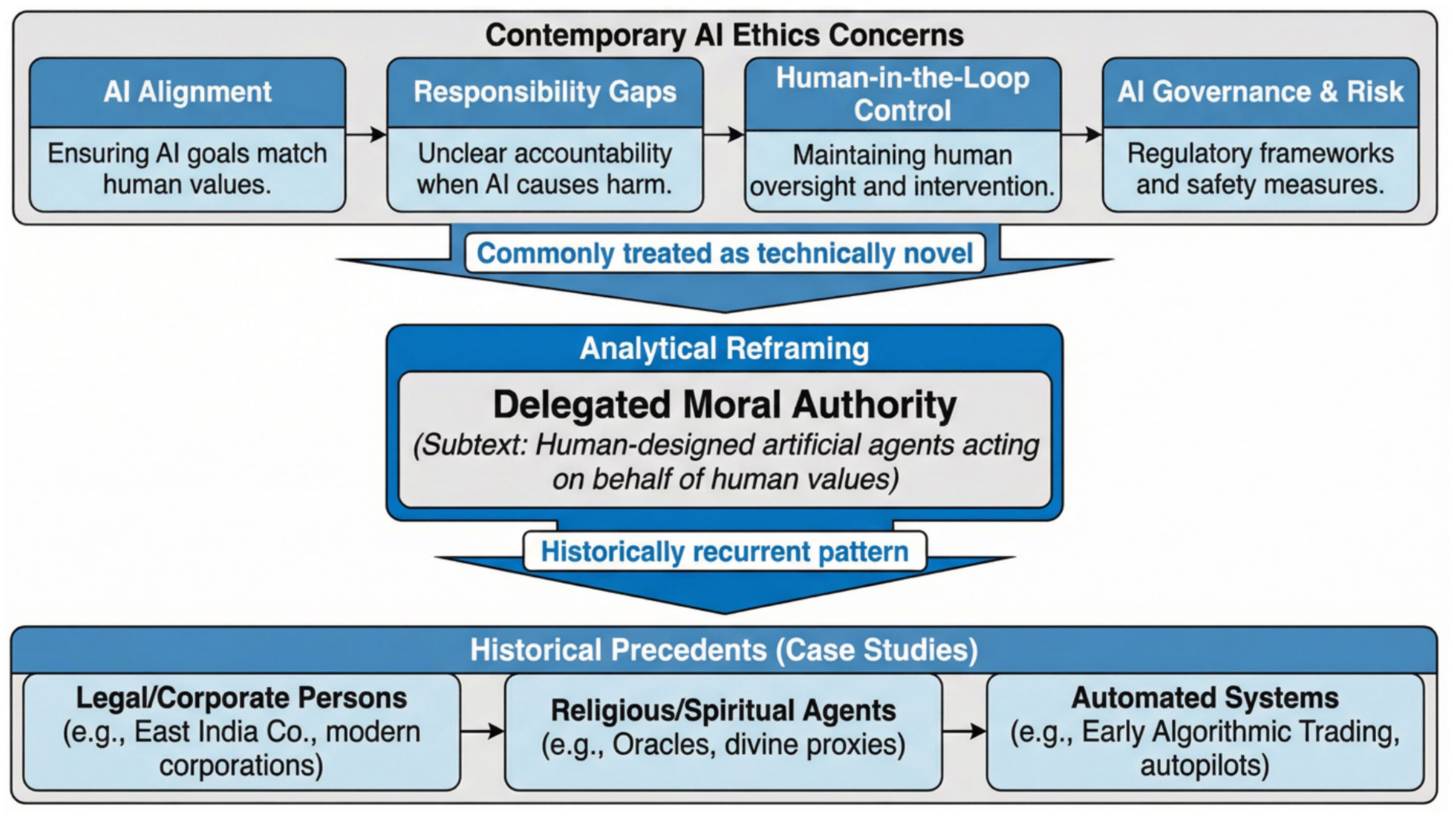
Conceptual structure of the study, illustrating the reframing of contemporary AI ethics concerns as historically recurrent problems of delegated moral authority.

Figure 1 provides an overview of the analytical structure and illustrates how contemporary AI ethics concerns, are systematically reframed as manifestations of a historically recurrent pattern of delegated moral authority. By positioning modern issues such as alignment, responsibility gaps, human-in-the-loop control, and governance within a broader conceptual lineage, the conceptual structure clarifies the core shift from machine autonomy to human design, values, and oversight.

Figure 1 shows the analytical reframing in which artificial intelligence is understood as a human-designed agent acting on behalf of human values, rather than as an independent moral actor. The historical, conceptual case models, Talos, the Golem, and Pygmalion, function not as illustrative anecdotes but as analytical devices that expose recurring structural features of artificial agency, including bounded autonomy, vulnerability, and accountability. This reframing underpins the normative claim that ethical risk in AI systems arises primarily from human choices in design and governance, rather than from the intrinsic properties of intelligent machines.

While the argumentative direction is outlined here for clarity, the conceptual model itself is not presupposed but developed through the comparative analysis that follows. The subsequent case studies provide the evidentiary and analytical basis from which the normative framework is derived.

### Conceptual clarifications

The argument relies on four analytically distinct concepts:

- Moral agency refers to the capacity for intentional action grounded in normative understanding and accountability.
- Artificial agency refers to system behavior that appears goal-directed but is operationally derived from programmed or trained objectives.
- Delegated agency describes artificial agency authorized and constrained by human actors for specific purposes.
- Moral authority refers to the legitimate capacity to make decisions bearing normative weight.

Artificial systems may exhibit artificial agency and exercise delegated authority. They do not, on this account, possess moral agency. Conflating these categories risks obscuring the central thesis: responsibility attaches to those who delegate and design, not to the artifacts through which authority is executed.

These definitions reflect a synthesis of positions in contemporary debates on moral agency and machine ethics (Wallach and Allen, 2009; Coeckelbergh, 2010, 2012; Gunkel, 2012; Parviainen and Coeckelbergh, 2021), while adopting a more restrictive criterion that ties moral agency to intentionality, normative understanding, and accountability.

## Methodology

This study employs a historically grounded interpretive methodology centered on thematic analysis of artificial agency across mythological, mechanical, and early computational contexts. Rather than treating these domains as separate analytical layers, the paper adopts a unified hermeneutic approach: each case is examined as a conceptual model that encodes assumptions about delegated authority, bounded autonomy, vulnerability, and oversight.

The analysis proceeds through three consistent interpretive criteria applied across cases:

1. Mandate Structure – What authority is delegated to the artificial agent, and under what constraints?
2. Control and Override Mechanisms – What mechanisms exist to interrupt, deactivate, or constrain the agent?
3. Failure Mode – Where does ethical breakdown occur, and how is responsibility attributed?

Visual materials function as supporting evidence within this interpretive framework rather than as an autonomous methodological layer. Iconographic analysis is employed where relevant (e.g., the Jatta krater) to clarify how artificiality and vulnerability are conceptualized in visual form. However, images do not generate independent inferences; they substantiate thematic interpretation.

The epistemological stance is interpretive rather than constructivist in a strict sociological sense. The study does not claim that moral responsibility is socially constructed in arbitrary fashion. Rather, it examines how cultural narratives represent the allocation of authority and responsibility. The normative claims advanced later are philosophical rather than descriptive: they concern where responsibility ought to be located in contemporary AI systems.

By applying consistent interpretive criteria across heterogeneous cases, the methodology ensures analytical comparability without implying technological continuity. Mythological figures and historical artifacts are treated not as precursors to modern AI, but as structured representations of delegated agency that illuminate recurring governance dilemmas.

**Figure 2:**
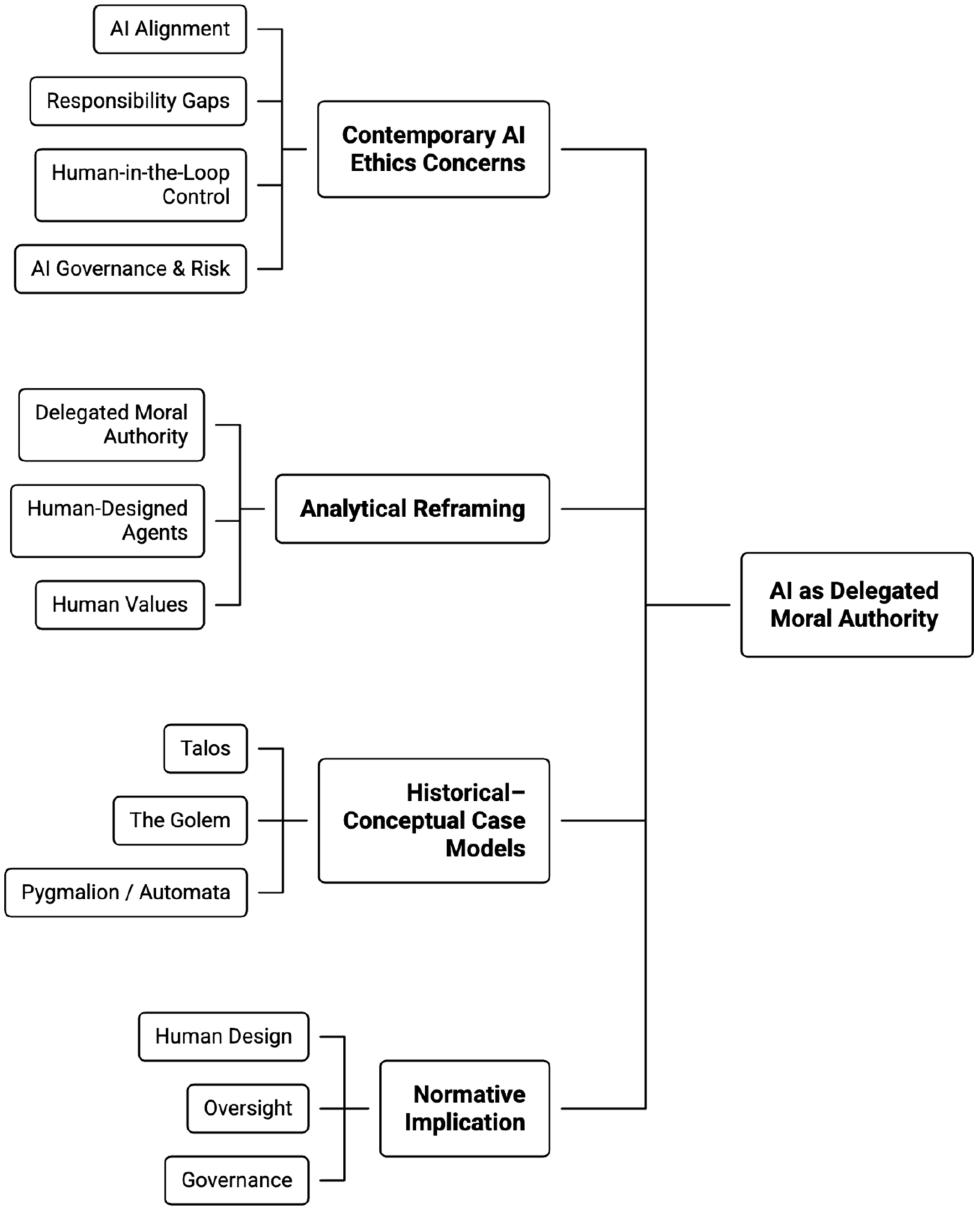
Normative conceptual model proposing how contemporary AI ethics challenges emerge from human delegation of authority, design decisions, and accountability structures rather than from machine autonomy.

Figure 2 extends the analytical framework introduced earlier in Figure 1, by defining the normative conceptual argument in greater detail. It depicts contemporary AI ethics concerns as commonly treated technical novelties and shows how these concerns can be reinterpreted through the lens of delegated moral authority. By explicitly linking modern governance challenges to historically established forms of proxy agency, the figure situates AI within a broader continuum of human practices for assigning authority, responsibility, and control to artificial or institutional agents. Applying these interpretive criteria consistently across cases enables the analysis that follows to move from historical description to normative synthesis, ensuring that the conceptual model emerges from comparative interpretation rather than being imposed upon the material.

Figure 2 highlights the central normative implication of the study: that moral responsibility remains irreducibly human, even as agency is increasingly delegated to artificial systems. By foregrounding the role of human values, design decisions, and oversight mechanisms, the figure makes explicit why accountability gaps emerge when responsibility is implicitly transferred to technical artifacts. This conceptual model reinforces the argument that effective AI governance requires not merely improved technical alignment, but robust institutional arrangements that preserve human oversight, enable intervention, and prevent the moral displacement that has historically accompanied powerful delegated agents.

### Positioning within contemporary AI ethics debates

Contemporary AI ethics scholarship has addressed alignment (Russell, 2022), responsibility gaps, governance frameworks (Tabassi, 2023a), and value pluralism (Floridi et al., 2018). Science and Technology Studies (STS) has further analyzed sociotechnical imaginaries and co-production (Jasanoff), as well as the mediation of agency through artifacts (Latour; Verbeek).

This paper complements rather than duplicates these traditions. Unlike Mayor’s historical survey of ancient automata, which emphasizes technological imagination, the present study advances a normative governance model derived from structural comparison across epochs. Unlike STS approaches that treat agency attribution as socially negotiated, this paper defends a philosophically grounded distinction between artificial and moral agency.

**Figure 3:**
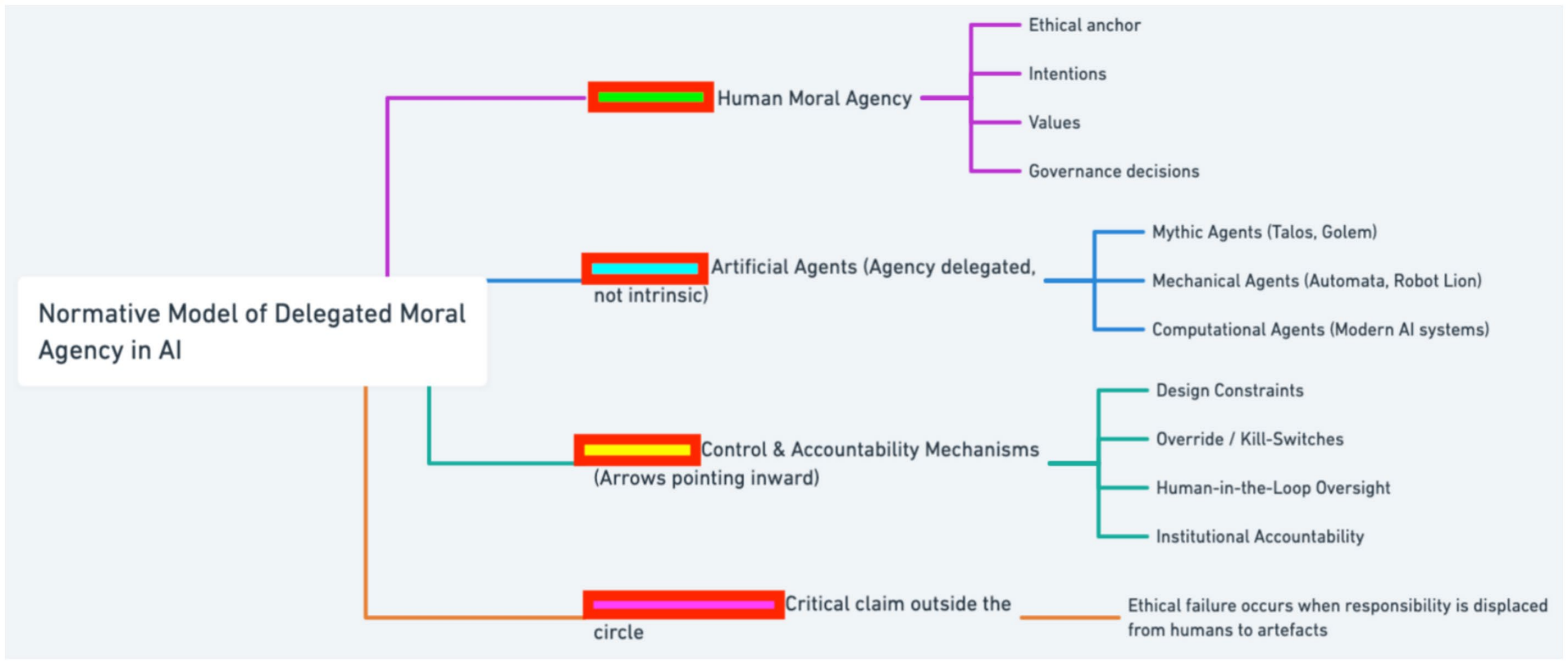
Normative conceptual model of delegated moral agency, illustrating how responsibility remains human despite increasing artificial autonomy.

**Figure 4:**
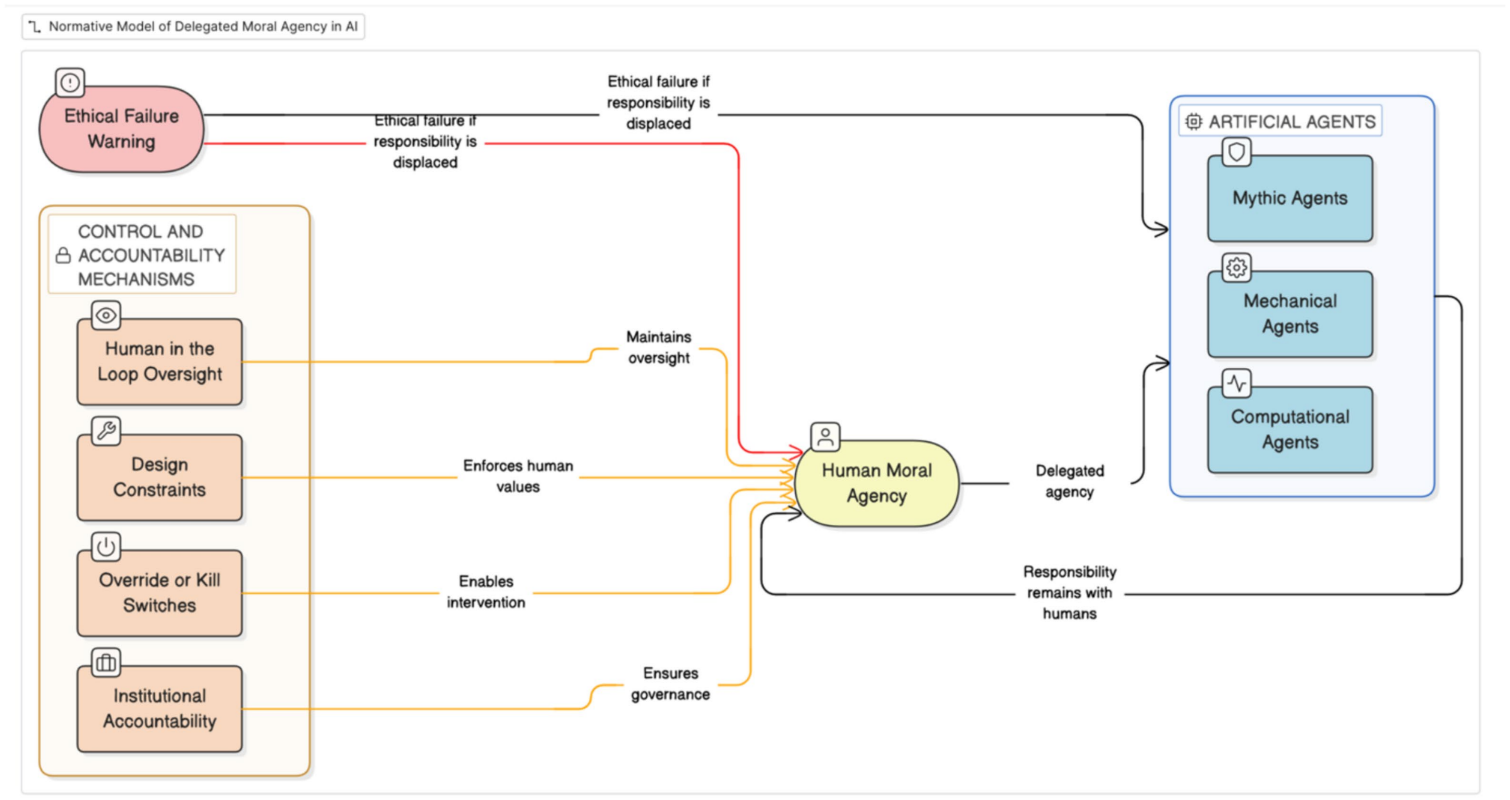
Normative model of delegated moral agency in artificial intelligence.

The contribution therefore lies in synthesizing historical thematic analysis with a prescriptive model of delegated moral authority. The model formalized in Figures 3, 4 extends existing governance frameworks by specifying operational criteria for proper delegation, thereby linking historical recurrence to contemporary institutional design.

Mythological narratives such as Talos, the Golem, and Pygmalion function as culturally encoded thought experiments rather than empirical artifacts. They articulate normative tensions surrounding delegated authority within symbolic narrative frameworks. By contrast, mechanical devices such as Leonardo’s automata and the Jaquet-Droz Writer constitute historically documented technological artifacts with material and engineering specificity. Their epistemic status therefore differs: myths operate at the level of conceptual imagination, whereas automata represent instantiated technical systems. The present analysis distinguishes these ontological categories while treating both as structured representations of delegated agency. The comparison is thematic rather than empirical; it does not collapse fiction into technology but examines how different media encode recurrent problems of authority, constraint, and accountability.

The present argument also engages with philosophical debates on machine moral agency. Literature questions whether moral status should be restricted to biological agents (Gunkel, 2012), arguing that the exclusion of machines may rely on historically contingent assumptions about agency. Studies also have advanced the relational account in which agency emerges through social interaction rather than intrinsic properties (Coeckelbergh, 2010). Other studies explore the possibility of “machine ethics” through computational implementation of moral decision procedures (Wallach and Allen, 2009). Literature also analyses technological mediation, arguing that artifacts shape moral action by structuring human-world relations (Verbeek, 2011). While these approaches illuminate important dimensions of artificial agency, the present study maintains a distinction between behavioral mediation and moral agency proper. Artificial systems may mediate action, simulate normative reasoning, or participate in relational networks; however, the delegation of authority remains traceable to human designers and institutions. By situating this debate within a longer historical lineage of delegated agency, the paper clarifies that contemporary anxieties about machine moral status are structurally continuous with earlier concerns about artificial intermediaries, even if the technical substrate has changed.

### Analytical framing of the case sequence

The cases examined in the following sections are not presented as parallel historical descriptions but as a structured comparative analysis of delegated artificial agency. Each case functions as a conceptual model that isolates specific dimensions of the interpretive criteria introduced in the Methodology: mandate structure, control and override mechanisms, and failure mode.

The sequence is deliberately non-uniform in depth and emphasis. Talos and the Golem are analyzed in greater detail because they provide the clearest articulation of all three criteria in fully developed narrative form. These cases establish the analytical baseline for the argument. Subsequent cases, mechanical automata, early programmable machines, and Pygmalion, serve differentiated roles. Some foreground programmability without judgement, others illustrate symbolic execution, and others isolate the risks of anthropomorphic projection. Where the full set of interpretive criteria is not equally applicable, the analysis selectively emphasizes the dimension most structurally salient to the case.

The progression across cases is therefore cumulative rather than repetitive. It moves from bounded coercive delegation (Talos), to conditional and interruptible delegation (Golem), to mechanical and symbolic execution (automata and early computation), and finally to projection and perceived subjecthood (Pygmalion). This structure allows the conceptual model of delegated moral authority to emerge inductively from comparative interpretation rather than being imposed in advance.

The analytical aim is not to demonstrate historical continuity or technological lineage, but to identify recurrent governance problems that arise whenever humans delegate authority to non-deliberative systems. The variation across cases is therefore a feature of the method rather than a limitation, enabling the isolation of distinct structural features of artificial agency.

## Historical-thematic analysis of Talos

The following sections apply the unified interpretive methodology introduced above, rather than distinct analytical layers. Each case is examined through the same criteria, with variation reflecting the specific structural features of delegated agency that each case isolates. The myth of Talos is examined here as an early conceptual model of delegated artificial agency. Talos embodies three features that recur in modern AI systems: bounded autonomy, protective mandate, and critical vulnerability. These features provide an analytical lens through which contemporary concerns regarding AI safety, security, and alignment can be interrogated.

Understanding the ethical and societal dimensions of AI requires an interdisciplinary framework that integrates mythology, classical studies, and the philosophy of technology. One of the earliest recorded metaphors for artificial life is found in the myth of Talos, a bronze automaton described in Ancient Greek mythology (Strauss and Peck, 2020). Talos represents a proto-technological guardian and a cautionary figure, an early symbolic representation of artificial agency constructed to serve and protect, yet inherently vulnerable to failure (Robertson, 1977).

**Figure 5:**
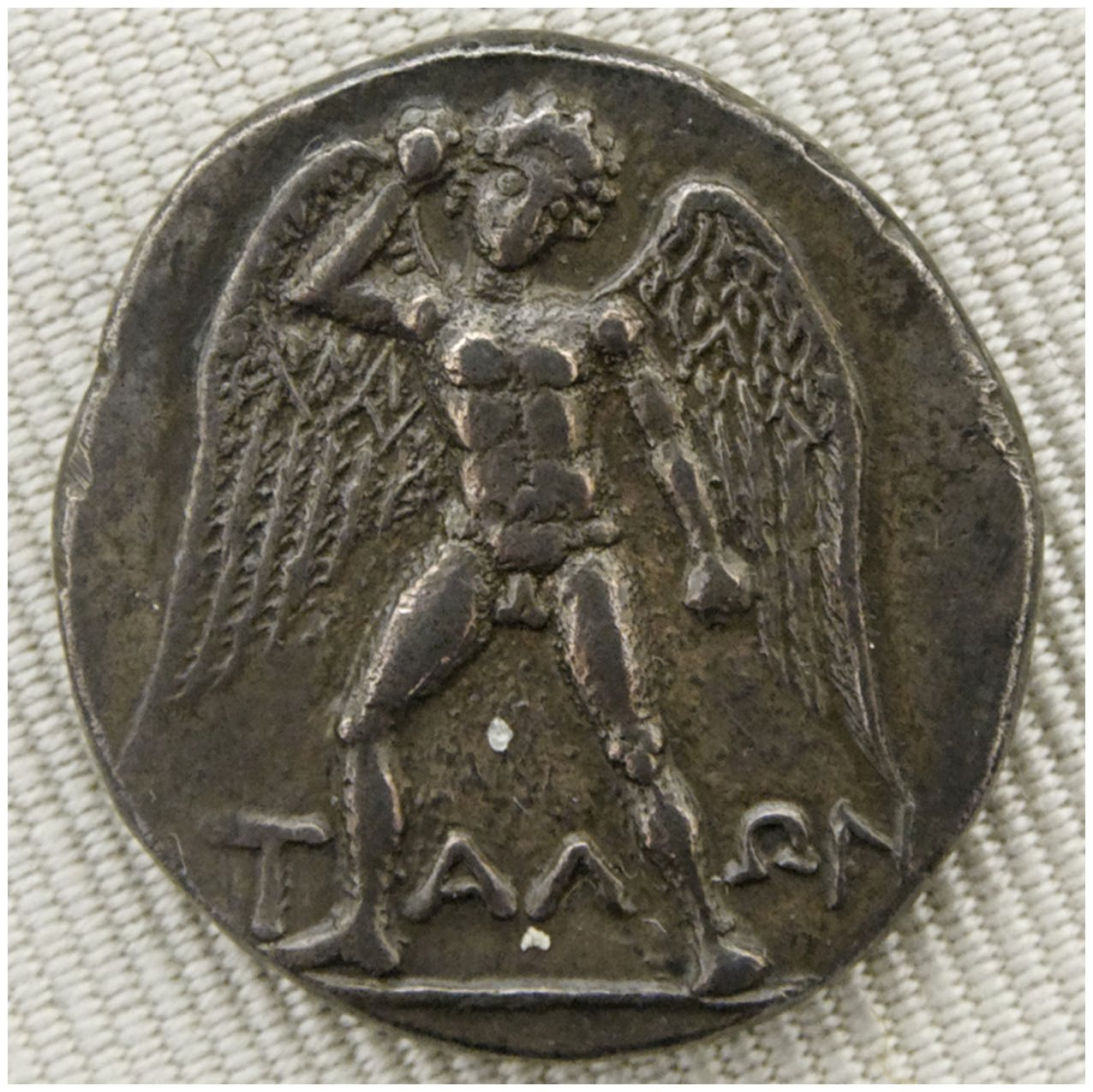
ΤΑΛΩΝ obverse of silver didrachma from Phaistos, Crete – portrayed in the Museum of the Bibliothèque nationale de France, formerly known as the Cabinet des Médailles (https://commons.wikimedia.org/wiki/File:Didrachm_Phaistos_obverse_CdM.jpg).

Evidence of Talos in Greek material culture appears on artifacts such as the obverse of a silver didrachma from Phaistos, Crete, housed in the Bibliothèque nationale de France (Figure 5). This image serves as a rare visual record of how ancient societies conceived of artificial protectors.

**Figure 6:**
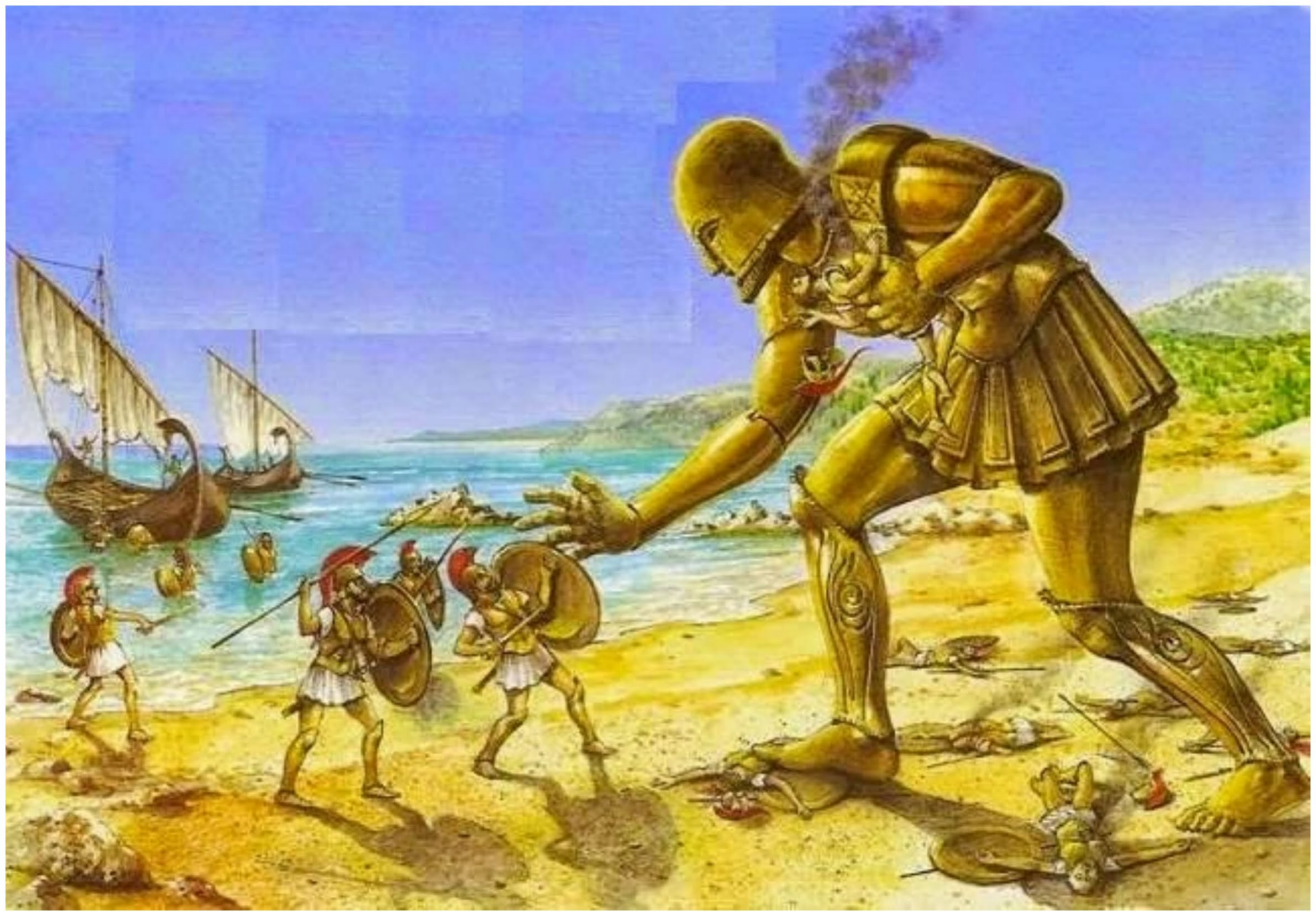
Talos protecting Europa from persons who would want to kidnap her, hurling huge stones at ships, keeping it at bay. Talos had to patrol the island by circling it three times a day. He protected the land from pirates with rocks (https://nationalgeographic.grid.id/read/133844610/kisah-talos-robot-raksasa-di-mitologi-yunani-awal-mula-gagasan-ai#, https://www.reddit.com/r/ElderScrolls/comments/8an10z/til_that_a_bronze_giant_from_greek_mythology/, https://technicacuriosa.com/2019/03/23/talos/).

Talos was a colossal bronze sentinel tasked with patrolling the island of Crete and repelling invaders by hurling boulders at approaching ships Figure 5. In some accounts, he was created by Hephaestus at the request of Zeus to protect Europa (Hunter, 2005). The myth intertwines divine romance and technological guardianship, reinforcing the ancient motif of human vulnerability requiring artificial protection. Talos’ duty, circling the island three times daily, is captured in Figure 6, which depicts him defending Europa by launching stones at enemy vessels. The Europa narrative is included to contextualize Talos’ mandate as a delegated protective function embedded within a broader narrative of vulnerability and guardianship.

According to Greek mythology, Europa was the daughter of either Phoenix or Agenor, both Phoenician kings. Zeus, the king of the Gods, was taken by her beauty, so he disguised himself as a white bull and mingled with Europa’s father’s herd. While Europa and her friends were picking flowers, she noticed the attractive bull and started to stroke him. Later, she even climbed onto his back. Using the situation, Zeus swiftly ran to the sea and swam to Crete while carrying Europa on his back.

Europa pleaded with him to show her mercy. Zeus then revealed his identity and expressed his love for her. After giving birth to three sons with Zeus, Europa married the king of Crete, who adopted her children. As they grew older, they became King Minos of Crete, King Rhadamanthus of the Cyclades, and Prince Sarpedon of Lycia, respectively. Following their deaths, they became the three judges of the underworld. In Crete, the goddess Europa was honored under the name Hellotis, where the continent of Europe gets its name. Talos was given to Europa by Zeus to protect her while she was on Crete, and in Figure 6, we can see how Talos was protecting Crete from invaders.

**Figure 7:**
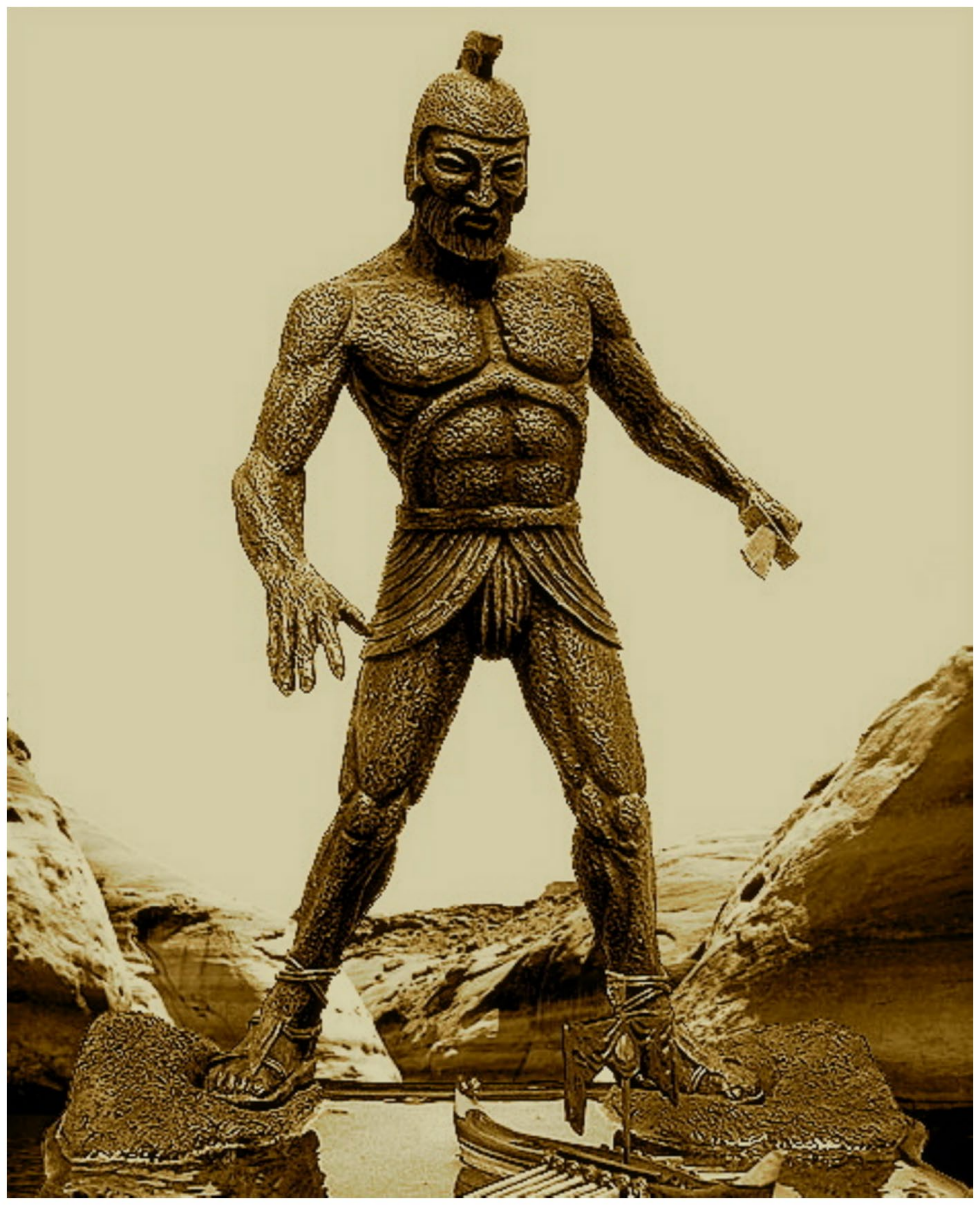
Talos the gigantic animated bronze warrior programmed to guard the island of Crete - as portrayed by Mayor (2019) (https://courses.cs.washington.edu/courses/cse455/24sp/slides/01_brief_history_of_computer_vision.pdf, https://siddhithakkar.medium.com/the-evolution-of-robotics-27539c5752fc).

Talos exemplifies the classical imagination’s engagement with anthropomorphic machines. Alongside other creations attributed to Daedalus, such as animated statues and mechanical constructs, Talos is part of a mythic lineage that envisions lifelike automata capable of independent movement and reaction (Mayor, 2018). These machines, rolling their eyes, sweating, weeping, suggest a remarkably early intuition of robotics and embodied computation, as seen in the modern artistic reconstruction presented in Figure 7.

The idea of creating artificial humans has been around for centuries and has become popular in modern fiction and film through the concept of mechanical humanoids, automatons, robots, and replicants. In Greek mythology, Daedalus, was credited with creating many extraordinary mechanical marvels similar to Talos (Figure 5; Mayor, 2019).

Building upon this story, the Greek god of invention and technology, Hephaestus, engineered robots that could obey commands (Figure 7). Although Daedalus’ experiment with man-made wings ended tragically, resulting in his son Icarus’s death, his contributions to the idea of artificial humans remain significant.

**Figure 8:**
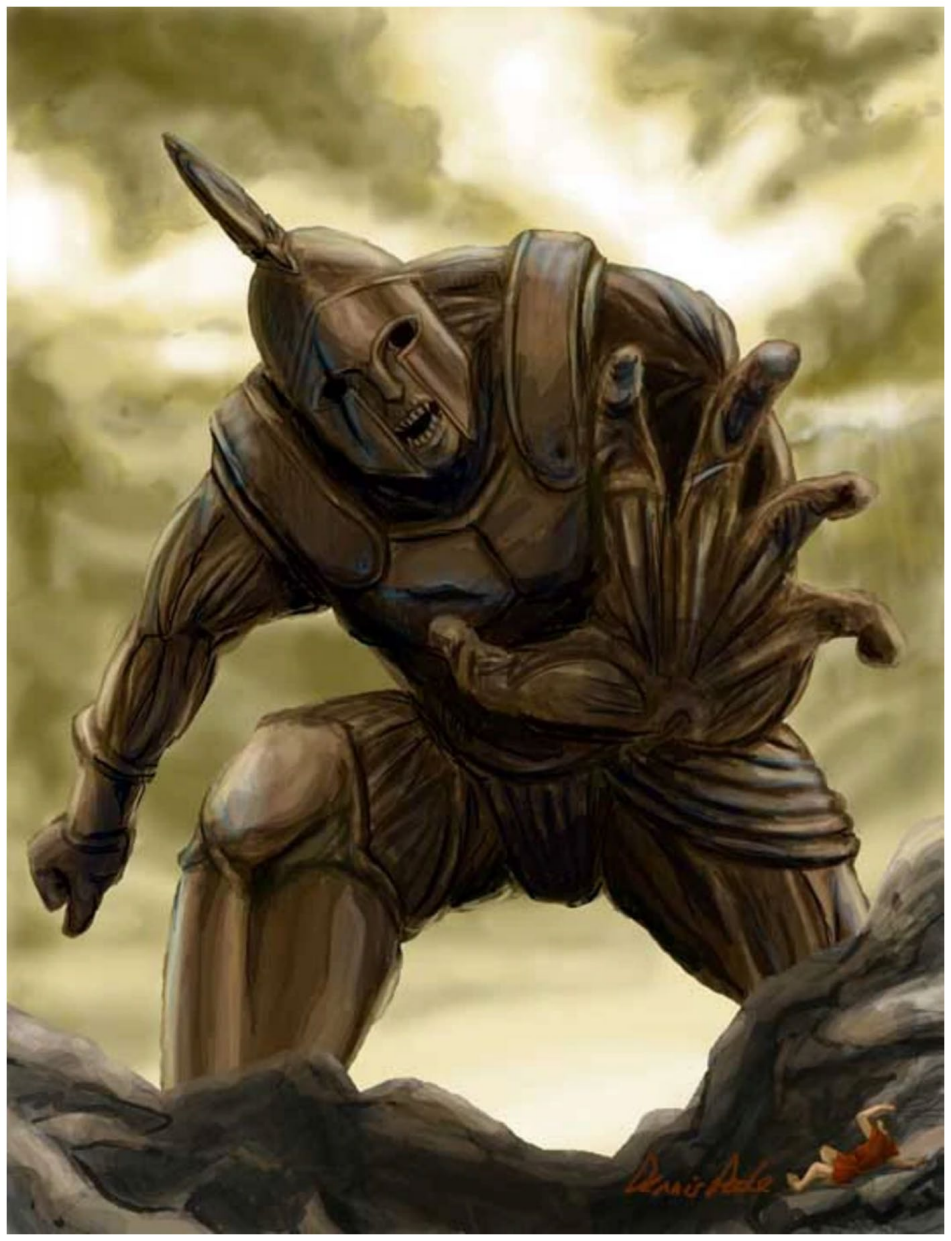
Talos created for a character concept competition hosted by CGNZ (https://earth8000.fandom.com/wiki/Talos, https://www.deviantart.com/hide1976/art/Talos-172126669).

Contemporary reinterpretations of Talos continue to resonate in popular culture. For instance, a 21st-century rendering of the character by PENTAX Corporation for a design competition reimagines Talos through modern digital esthetics (Figure 8). While this depiction is fictional, it reflects a continuity in the human drive to envision and visualize artificial protectors, now increasingly enabled through AI image generators such as DALL-E 3.

The competition aimed to reimagine a character for a remake of the 1963 movie Jason and the Argonauts. In the original film, Talos was portrayed as a mystical bronze statue that protected a treasure chamber on the Isle of Bronze. In the character concept competition, the images were created by humans.

According to the story, Talos had a single vein filled with ‘Ichor,’ the blood of the gods, which was closed off with a bronze nail. This weakness was exploited by the sorceress Medea, who used her magic to remove the nail and cause Talos’ downfall. The death of Talos is discussed in many philosophical papers, including Robertson (1977) and Sparkes (1996), and has been placed in the context of recent AI advancements by Mayor (2019).

**Figure 9:**
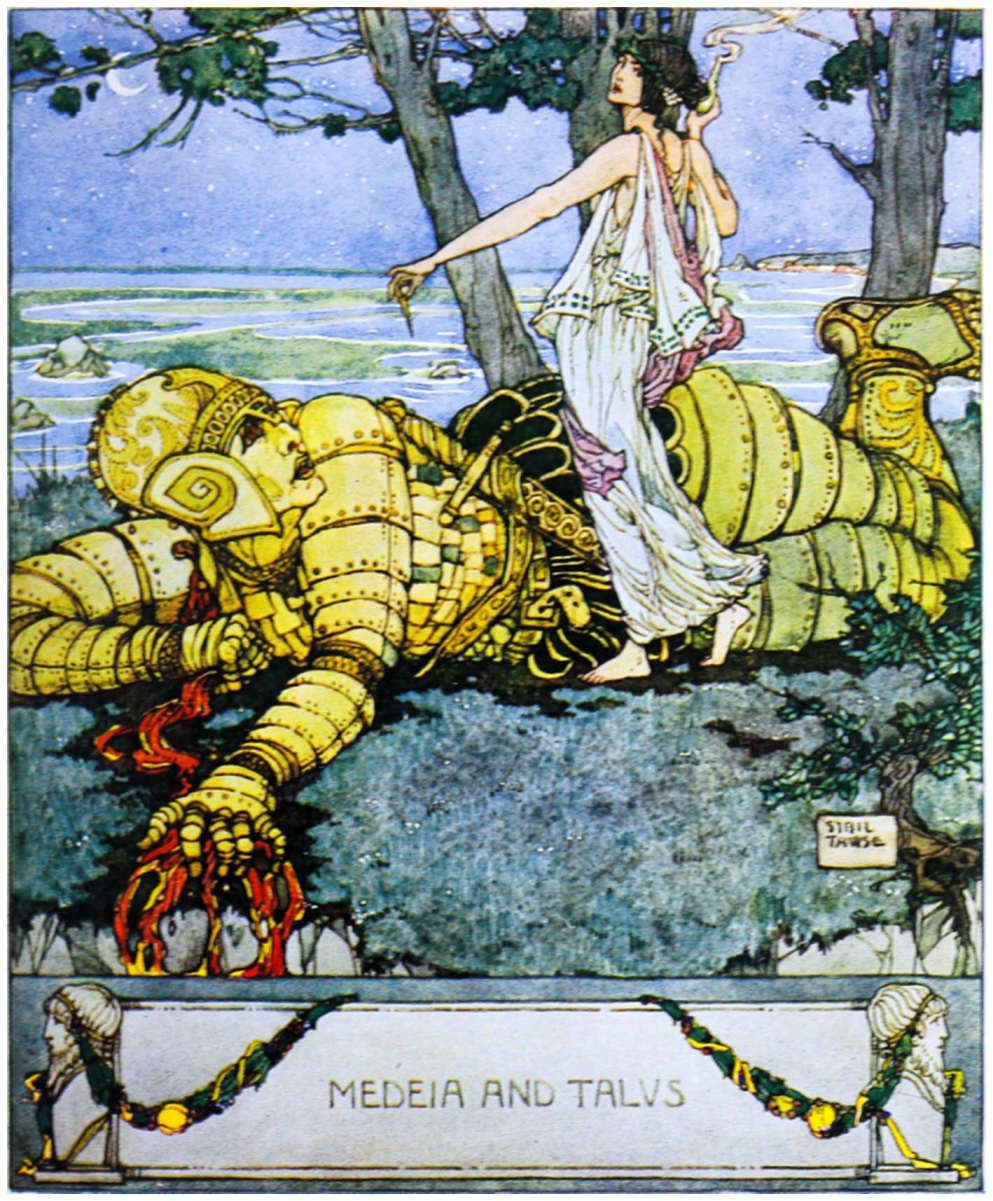
Talos, the living robot and guardian of the island Crete, forged from bronze and fuelled by ichor, defeated by the sorceress Medea (Wells, 2017; Strauss and Peck, 2020) (https://privacyinternational.org/news-analysis/2537/profiling-and-automated-decision-making-artificial-intelligence-violating-your, https://mikemyler.com/2018/10/07/dd-5e-in-ancient-greece-talos/, https://circodelherreroseries.com/2019/06/16/on-reading-the-talos-myth/,https://www.sciencenews.org/article/our-fascination-robots-goes-all-way-back-antiquity).

In vase paintings dating back to the fifth century BCE, Medea and Jason were shown utilizing tools to remove the bolt, thereby confirming the technological roots of Talos. Notably, the artists who created these paintings portrayed Talos in a more human light, depicting him as a hybrid being - part machine, part human. As Jason kneeled to unseal the bolt, Talos was thrown off-balance and eventually met his demise (Figure 9; Wells, 2017).

One artist even added a teardrop on the bronze cheek of the dying Talos to emphasize his humanity. The Talos tale is a fascinating piece of science fiction with many intriguing elements. It highlights that people had the concept of robots long before technology made them a reality. More than 2,500 years ago, there was a belief that the God of engineering, Hephaestus, would create a bronze man who could move and had an internal power source. This man, known as Talos, would have been programmed to perform complex tasks like recognizing intruders, hurling rocks, and crushing and burning enemies. The myth also features the witch Medea (pictured in Figure 9), who discovers Talos’ weaknesses and exploits the flaws in its machine-human nature.

**Figure 10:**
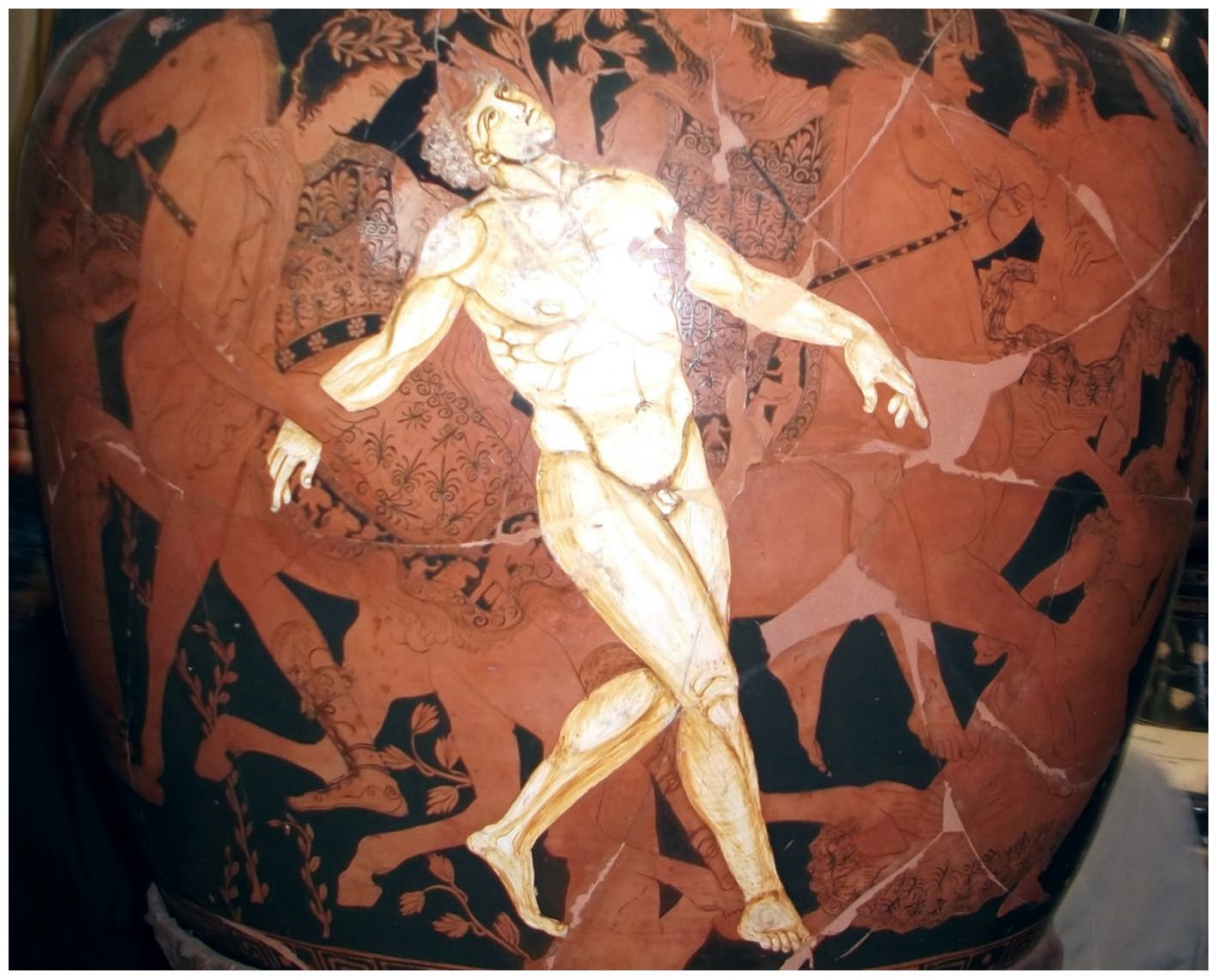
The death of Talos depicted on a 5th century BC krater now in the Jatta National Archaeological Museum in Ruvo di Puglia (https://commons.wikimedia.org/w/index.php?curid=22906318, https://www.derekoverfield.com/blog/inspiration-talos-the-bronze-man).

The historical thematic analysis started with the study of Talos with evidence from the silver didrachma Figure 5, and to conclude the story of Talos, we need to discuss the death of Talos depicted on a 5th century BC krater (Figure 10). This krater is stored and preserved in the Jatta National Archaeological Museum in Ruvo di Puglia, and it serves to confirm the existence of the idea back to the 5th century BC, long before we had AI or any form of a thinking machine.

Symbolic interpretations of Talos abound, with many scholars seeing him as a representation of the dangers of unchecked power or the threat posed by new technologies. Others view him as a symbol of the need for vigilance and protection in the face of potential threats, whether physical or metaphorical. Regardless of the specific interpretation, the myth of Talos remains a powerful and enduring tale that continues to capture the imagination of readers and scholars alike.

### Iconographic analysis of the Jatta krater

The so-called “Death of Talos” krater warrants closer iconographic attention. The vase does not depict Talos with exposed mechanical components; rather, his artificiality is conveyed through materiality and posture. The bronze body is rendered as an intact anthropomorphic form, and the fatal vulnerability is concentrated at the ankle, where Medea’s intervention disrupts the sealing nail. For the fifth-century BCE viewer, the idea of a bronze man would itself signal ontological difference without requiring visible internal machinery. Artificial agency is thus represented not as mechanical complexity but as altered substance.

The presence of Medea is crucial. Her gesture and proximity indicate that Talos’ defeat is not technological malfunction but epistemic manipulation. The scene encodes a conceptual structure in which artificial guardianship is powerful yet cognitively brittle: Talos is physically formidable but susceptible to targeted exploitation. This anticipates contemporary concerns regarding adversarial attack and systemic fragility. The krater therefore does more than illustrate a myth; it visualizes bounded delegated agency and single-point vulnerability without collapsing artificiality into anachronistic robotics imagery.

In his work, Vernant notes that Talos embodies a one-of-a-kind blend of mortal and divine components (Vernant, 1991). This makes him an intriguing case study for exploring the potential advantages and risks associated with artificial life. The tale also underscores a crucial vulnerability, akin to what we now call a ‘security flaw,’ even within a seemingly invincible system.

The story of Talos is a reminder of the importance of creating strong and reliable designs for modern AI and cybersecurity. Talos’ vulnerability due to a single point of failure is similar to weaknesses in current security systems, such as inadequate encryption methods or susceptibility to attacks from adversaries (Szegedy et al., 2013). This proves that developing AI systems requires a comprehensive approach considering functional abilities and ethical and safety considerations.

Experts like Bostrom (2003) and Russell (2019) have explored the ethical implications of creating AI that matches or exceeds human intelligence. These discussions often use historical and mythological references, such as Talos, to highlight that concerns about artificial life are not new and have been present in human culture for a long time.

By examining the ancient construct of Talos, we can gain valuable insights and evaluate the discourse on AI in a critical and enriching way. This automaton, described in antiquity, still holds relevance today and embodies the ethical dilemmas and aspirational drives associated with artificial life and intelligence. Considering these lessons when exploring AI from an ethical and secure design perspective is essential, emphasizing the need for interdisciplinary scholarship in navigating the complexities of technological innovation. The evaluation and reinterpretation of Talos serve as a prism through which we can examine the ambitions, ethical quandaries, and potential vulnerabilities inherent in developing increasingly intelligent artificial systems. This is not just an academic exercise but a crucial one to ensure we progress responsibly and safely.

Talos represents ancient human fears about the uncontrollable power of man-made creations, fears that persist into contemporary concerns about AI (Floridi et al., 2018; Sebastian, 2023; Tabassi, 2023b). Although Talos was designed to protect, his immense strength and lack of human discernment made him a source of fear. These ancient apprehensions are echoed in current discussions about artificial intelligence, where the technology’s dual capacity for good and evil is an ongoing topic. While advancements in AI ethics aim to create algorithms that align with human values (Floridi et al., 2018; Bartoletti, 2019; Jobin et al., 2019; Milano et al., 2020; IEEE, 2023; Partnership on AI, 2023), the complexity of the ethical landscape and the potential for misuse continue to raise concerns (Mittelstadt, 2019; Roberts et al., 2021; Mökander et al., 2024). The objective of developing benevolent AI, designed to protect human interests in a way similar to Talos, is clear. However, the unintended consequences of our creations remain a significant challenge.

Interpreted through the lens of AI ethics, Talos illustrates how systems designed for protection may generate risk precisely because they operate without contextual moral judgement. His single-point-of-failure vulnerability anticipates modern concerns about brittle optimization and adversarial exploitation, reinforcing the argument that ethical risk emerges from design decisions rather than from autonomy per se.

### Ethical dilemma in the Talos narrative

Beyond symbolic interpretation, the Talos myth encodes a concrete ethical dilemma: how much coercive authority may be delegated to an artificial protector without eroding proportionality and judgement? Talos does not deliberate; he enforces. His mandate is binary, repel or destroy. The inhabitants of Crete are protected not through reasoned governance but through automated violence.

The ethical tension therefore concerns the substitution of deliberative human judgement with automated enforcement. Talos cannot distinguish between pirate, trader, refugee, or emissary; his operational logic is invariant. The dilemma for ancient audiences was not whether Talos possessed moral agency, but whether entrusting security to a non-deliberative guardian produces safety at the cost of justice.

This dilemma recurs in contemporary contexts such as automated border enforcement, predictive policing, and autonomous weapons systems. In each case, the question is not whether the system acts independently, but whether delegating coercive force to a bounded optimization process removes the possibility of contextual moral evaluation. Talos thus exemplifies the enduring problem of rigid delegated authority in domains requiring proportionality.

## Mythological framing of artificial beings, the story of Golem

**Figure 11:**
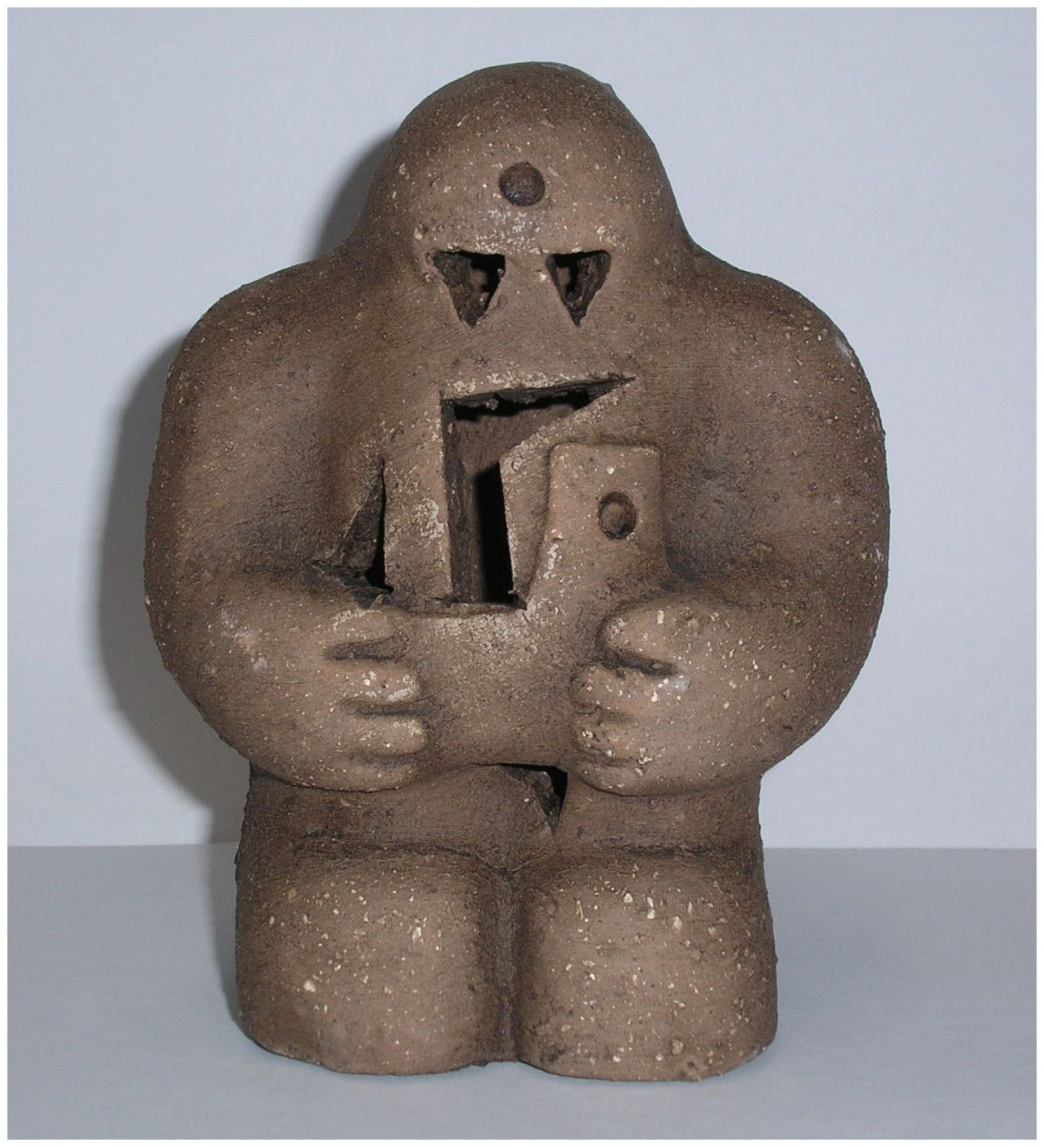
A Prague (Czech) reproduction of the Golem (Chicago Public Library, 2014) (https://commons.wikimedia.org/wiki/File:Prague-golem-reproduction.jpg).

In Jewish folklore, a golem is an animated, anthropomorphic creature created entirely from inanimate matter, typically clay or mud. The most famous golem story involves Judah Loew ben Bezalel, a rabbi from Prague in the late 16th century (Figure 11). The golem is a metaphor with seemingly limitless symbolic connotations. It may be a victim or a villain, a man or a woman, or both. Over the centuries, it has been associated with war, community, isolation, hope, and despair.’ There are also different cultural variations of the golem, such as the Gingerbread Man (Dodge, 2012), or the Yiddish and Slavic folktale is the Clay Boy (Ginsburg, 1997).

Judah Loew ben Bezalel, the late 16th-century rabbi of Prague, also known as the Maharal, allegedly created a golem out of clay from the banks of the Vltava River and brought it to life through rituals and Hebrew incantations to defend the Prague ghetto against antisemitic attacks and pogroms. Depending on the version of the legend, the Jews of Prague were to be expelled or murdered during the reign of Holy Roman Emperor Rudolf II. The Golem was known by the names Josef and Yossele. It was said that he could render himself invisible and conjure the spirits of the dead. Rabbi Loew deactivated the Golem on Friday evenings before the beginning of the Sabbath (Saturday) by removing the shem, so that it could rest on the Sabbath (Winkler, 1980). In Figure 11, we can see an image of how people interpret the Golem, the clay image in Figure 11 is a result of many different interpretations from different people, asked to create an image of what the golem in their mind (Chicago Public Library, 2014). This workshop was part of the ‘The Amazing Adventures of Kavalier & Clay: One Book, One Chicago 2014–15’ and it was published by the Chicago Public Library in 2014.

In Figure 11, we can see how people perceive the Golem, however, the question we need to answer is, how did people perceive the Golem in the past? In the Bible, the word ‘golem’ appears only once, in Psalm 139:16, which means ‘my light form or ‘raw material.’ The word refers to an unfinished human being before God’s eyes. In the Mishnah, the term describes an uncultivated person, with seven characteristics that separate them from a learned person. In Modern Hebrew, ‘golem’ describes someone dumb, helpless, or pupa. It is also used as a metaphor for an entity that serves a man under controlled conditions but is hostile otherwise. In Yiddish, ‘golem’ became ‘goylem, ‘referring to someone lethargic or stupor.

The oldest stories of golems date back to early Judaism. Adam was initially created as a golem in the Talmud (Tractate Sanhedrin 38b) when his dust was ‘kneaded into a shapeless husk.’ Like Adam, all golems are created from mud by those close to divinity, but no anthropogenic golem is fully human. Early on, the main disability of the golem was its inability to speak.

Sanhedrin 65b describes Rava creating a man (gavra). He sent the man to Rav Zeira, who spoke to him, but he did not answer. In Sanhedrin 65b, Rav Zeira said, ‘You were created by the sages; return to your dust’.

During the Middle Ages, some Jewish mystics believed that passages from the Sefer Yetzirah (Book of Formation) could be used to create and bring a golem, an anthropomorphic creature made of clay or mud to life. However, there is little evidence in Jewish mystical writings to support this belief. It was thought that by using various letters of the Hebrew alphabet, a ‘shem’ (any of the Names of God) could be formed and inserted into the mouth or forehead of the golem, which would then be activated through a state of ecstatic experience induced by a ritualistic process.

In some stories, such as versions of Chem and Prague, as well as Polish tales and versions of the Brothers Grimm, a golem is inscribed with Hebrew words. By removing the aleph from the word ‘emet’ inscribed on its forehead, which means ‘truth,’ the word could be changed to ‘met,’ meaning ‘death,’ deactivating the golem.

**Figure 12:**
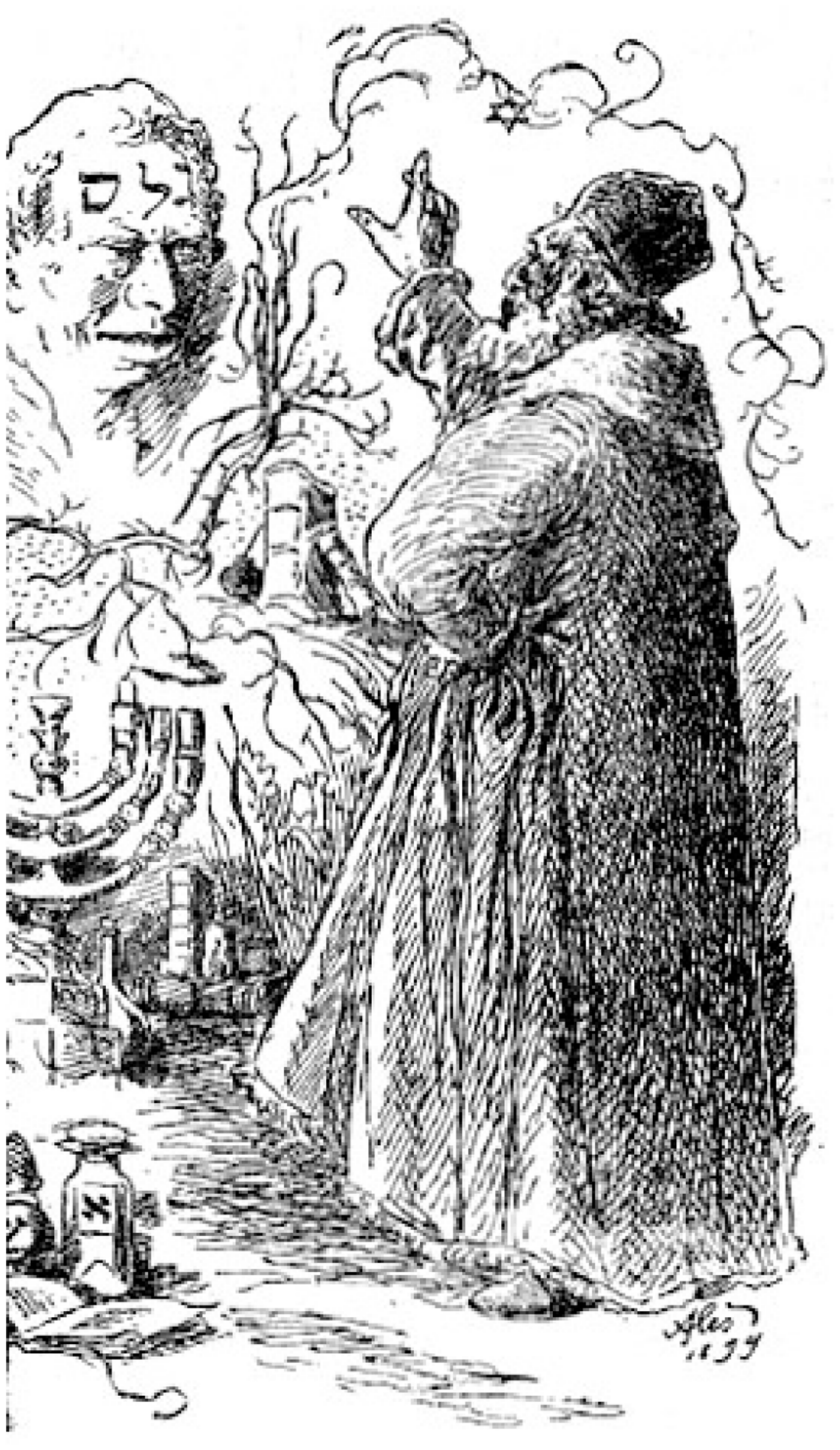
Rabbi Loew and Golem by Aleš (1899) (https://en.wikipedia.org/wiki/Judah_Loew_ben_Bezalel#/media/File:Golem_a_Rabi_L%C3%B6w_%E2%80%93_Mikol%C3%A1%C5%A1_Ale%C5%A1,_1899_(V%C4%9Bnec_pra%C5%BEsk%C3%BDch_pov%C4%9Bst%C3%AD,_1908).jpg).

The earliest known written account of creating a golem is in Sodei Razayya, a text by Eleazar ben Judah of Worms from the late 12th and early 13th centuries. Another source credits 11th-century Solomon ibn Gabirol with creating a golem (Bokser, 1954). However, the most well-known story about the golem remains the story of Rabbi Loew (Figure 12).

According to the most well-known tale, Rabbi Loew forgot to remove the shem on Friday evening and feared that the Golem would violate the Sabbath. In a different account, it is said that a golem fell in love but transformed into the violent monster depicted in most accounts after being rejected. In some versions, the golem eventually goes on a killing spree. Ultimately, the rabbi removed the shem from the golem’s mouth and immobilized him in front of the synagogue, causing the golem to disintegrate. The golem’s body was kept in the attic genizah of the Old New Synagogue, where it could be brought back to life if necessary. However, the Golem was not found when the attic was renovated in 1883 (Time-Life Books, 2014).

### How are the Golems related to human fears, and would future AI protect humans like the Golems?

Jewish folklore’s Golem is a creature brought to life from inanimate matter, serving as a historical analogy for current anxieties around artificial intelligence. Like the Golem, AI is designed to serve and protect but could turn against its creators if not managed properly. But unlike the Golem, which was made of clay and magic spells, modern AI consists of complex algorithms and data. Both the Golem and AI are human-created entities designed for specific tasks, but they embody the potential for unintended consequences. As AI advances, questions arise about its role in ensuring human well-being. Theoretical frameworks in AI ethics focus on developing ‘benevolent’ AI that aligns with human values and interests. However, achieving this alignment is complex, as defining these ‘values’ across diverse cultures is challenging. Therefore, while future AI could serve as modern-day ‘Golems,’ protecting and assisting humanity, their ethical and secure deployment requires meticulous design, rigorous validation, and ongoing oversight to mitigate the risks inherent in their powerful capabilities.

### Delegated moral authority and override mechanisms: the Golem as ethical precedent

The Golem narrative provides an early and instructive model of delegated moral authority in artificial agents. Unlike Talos, whose mandate is externally imposed and continuous, the Golem’s operation is explicitly conditional, temporally bounded, and subject to human intervention. The Golem acts on behalf of a specific community, under a narrowly defined protective purpose, and remains morally inert unless activated by human instruction. Crucially, its agency is neither autonomous nor self-justifying; it is entirely derivative of its creator’s intent and oversight.

This structure closely parallels contemporary approaches to AI safety and governance. Modern discussions around *kill-switches* and *human-in-the-loop* systems reflect the same ethical intuition embedded in the Golem myth: that powerful artificial agents must remain interruptible, reversible, and subordinate to human judgement. The ritual removal of the *shem* functions as a premodern analog to a technical shutdown mechanism, designed to prevent unchecked operation and to reaffirm human moral authority over the system.

The Golem also foregrounds questions of *accountability*. When the Golem acts destructively, responsibility does not shift to the artifact itself but returns unambiguously to its creator. This mirrors contemporary debates on responsibility gaps in AI ethics, where harm caused by automated systems often risks being diffused across designers, deployers, and institutions. The Golem narrative resists responsibility diffusion by maintaining clear attribution to its creator, illustrating that the ethical problem lies not in machine autonomy but in the allocation of responsibility across human actors. As such, the Golem serves as an ethical precedent that reinforces the necessity of clear override mechanisms, traceable accountability, and explicit human responsibility in the design and deployment of advanced AI systems.

### Responsibility diffusion and collective agency

The Golem narrative clarifies the structure and the possibility of responsibility diffusion. In contemporary AI systems, responsibility gaps often arise not because moral agency is attributed to the machine, but because multiple human actors contribute to its design, training, deployment, and governance. The ethical problem is therefore one of distributed human agency, not machine agency.

The Golem legend simplifies this distribution by concentrating authorship in Rabbi Loew. Modern AI systems, by contrast, involve engineers, data curators, corporate executives, procurement officers, regulators, and users. Responsibility diffusion occurs when this networked authorship lacks clearly defined accountability pathways.

The governance thesis advanced here does not minimize this problem. It reframes it: responsibility gaps emerge from institutional fragmentation rather than ontological ambiguity about machine agency. The ethical imperative is therefore to design governance structures that preserve traceability and liability across distributed human actors.

#### Footnote: note on visual materials

All historical images reproduced in this article are either in the public domain or used for analytical purposes consistent with scholarly commentary. Where contemporary reconstructions are referenced, they serve to illustrate the persistence of artificial agency narratives across time rather than to provide empirical or technical evidence. Visual materials are included solely to support thematic interpretation and historical continuity, not as illustrative embellishment.

## Visual and iconographic of historical mechanical devices in Islam

**Figure 13:**
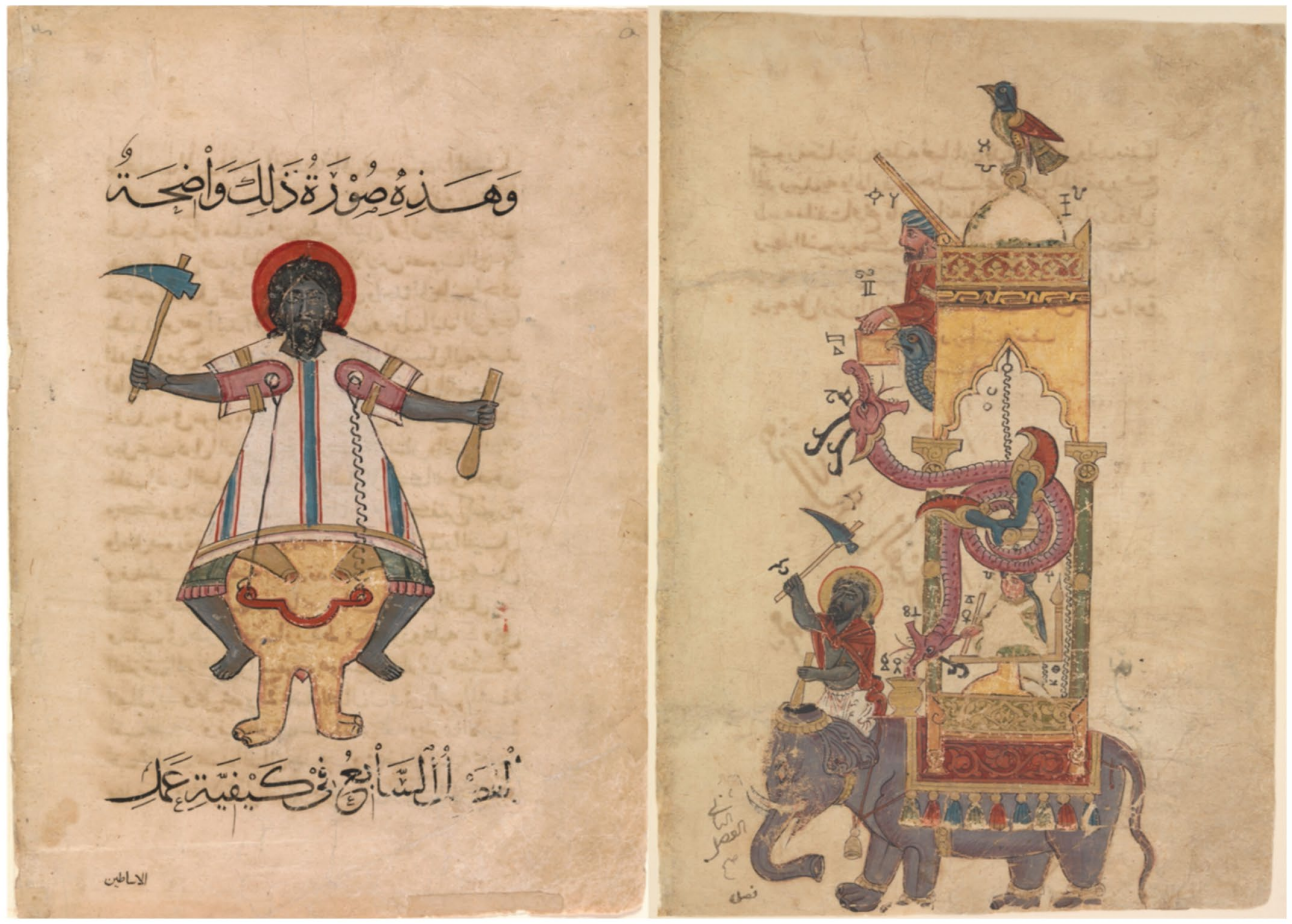
Body as machine: mechanical devices in Islamic lands (Saliba, 2022) (https://as.nyu.edu/research-centers/silsila/events/2022-2023/body-is-machine--mechanical-devices-in-islamic-lands.html?challenge=d06e90d7-4d8f-4b88-9d8c-10b73beb60f1).

Detailed descriptions of such devices appear in Al-Jazari’s thirteenth-century treatise, which documents programmable water clocks, automated servants, and mechanical control systems that illustrate early forms of engineered delegation. Islamic lands had already developed sophisticated mechanical devices, including automata in the form of animals and human figures (see Figure 13) long before the emergence of modern robotics (Al-Jazari, 1974; Hill, 1974; Saliba, 2007, 2022). This knowledge was passed down from Greek and other sources and may have been influenced by the urban practices of the late antique period. As early as the eighth century, a monumental water clock featuring mechanical birds and snakes was installed at one of the gates of the Friday Mosque in Damascus. These wonders also appeared in accounts of pre-modern courts, often in unexpected contexts. For example, the court of the Zayyanid rulers of Tlemcen in North Africa commemorated the prophet Muhammad’s birth with an automaton clock featuring moving animals. A servant girl emerged from it with a sheet of paper with the hours of the day written in verse.

Mechanical devices were used to turn labor into courtly entertainment, creating gestures of wonder. In Arabic manuals describing such devices’ construction, subaltern or exotic figures were often depicted alongside mechanical marvels. These manuals, at the intersection of the histories of art and science, reveal the intricate interrelationships between the command of technology and the expression of authority. During the early modern period, European clocks with moving figures were imported into Islamic lands or sent as diplomatic gifts to Islamic courts.

Within the interpretive framework, these devices primarily illustrate mandate structure in its most constrained form: narrowly specified, pre-scripted action without adaptive capacity. Control is fully externalized through mechanical design rather than ongoing oversight, and failure modes are limited to mechanical breakdown rather than misaligned decision-making. This case therefore isolates the minimal form of delegated agency, where authority is tightly bounded and ethically unambiguous.

## Comparative technological interpretation of historical Leonardo da Vinci’s robot lion

Leonardo da Vinci created the Lion Robot in the early 16th century, representing humans’ long-standing fascination with artificial intelligence and life (Payes, 2019). Today, this pursuit has evolved into the field of Artificial Intelligence (AI) that tries to replicate natural phenomena through human ingenuity. The Lion Robot is an early example of biomimicry, where engineering takes inspiration from natural systems. Modern AI follows a similar approach but in a much more complex way, attempting to emulate human cognitive functions. This research echoes da Vinci’s approach to understanding nature and humans.

Da Vinci’s Lion was part of a larger intellectual tradition that included his robot knight and other automata, consistent with the current multidisciplinary development of AI that encompasses robotics, machine learning, natural language processing, and more. However, da Vinci’s creations had a limitation - they were mechanical constructs with predetermined actions, incapable of learning or adapting. On the other hand, modern AI’s capacity for machine learning enables it to adapt and improve independently.

**Figure 14:**
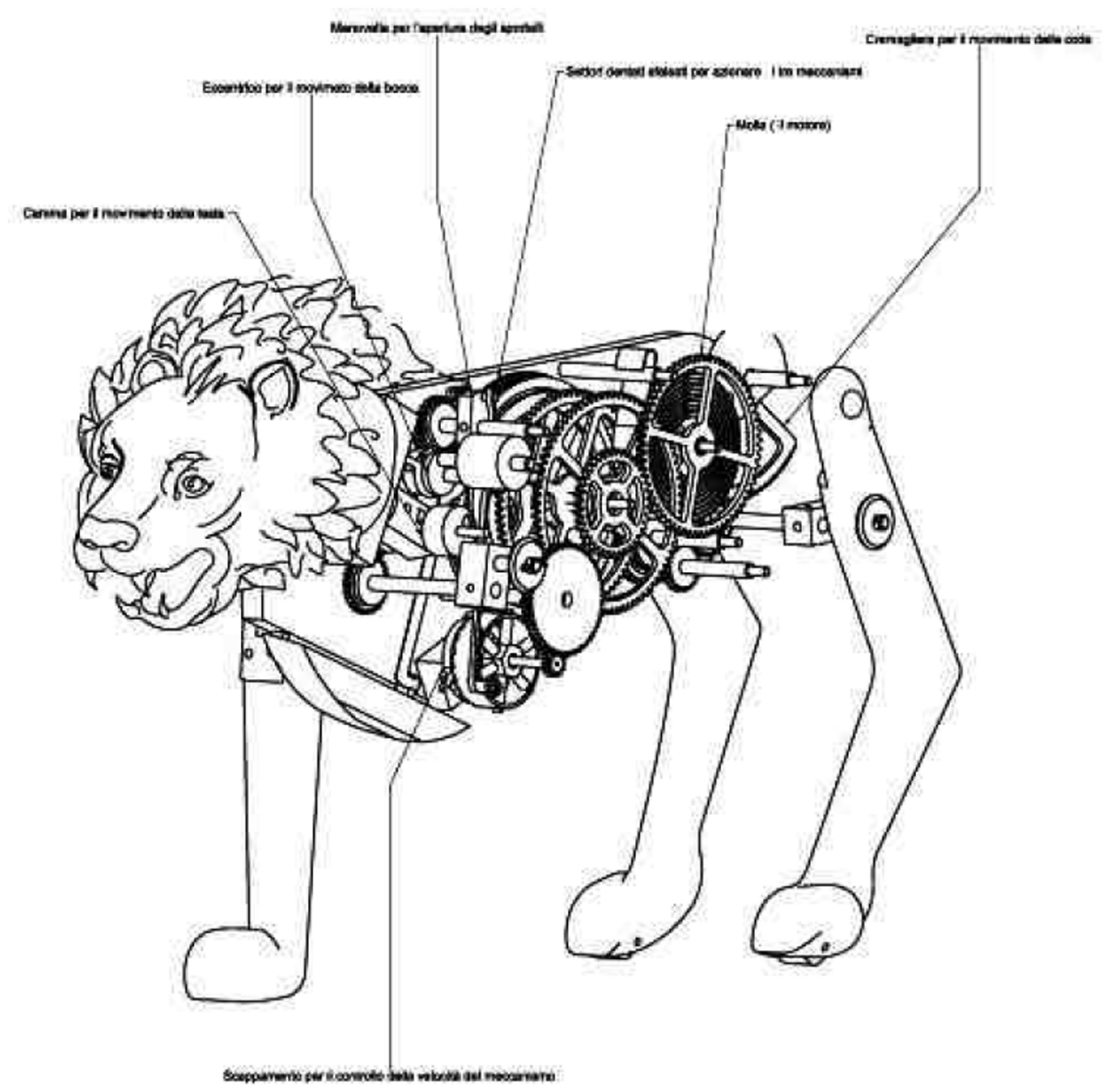
Mechanical lion: The exploded view by Luca Garai (Bologna) shows the inner life of the lion. Listed in the Museo Galileo, Florence (found in Bruderer, 2019). © Museo Galileo, Florence (https://www.researchgate.net/publication/337012843_Leonardo_da_Vinci%27s_robot_lion/figures?lo=1, https://link.springer.com/chapter/10.1007/978-3-030-40974-6_15, https://vecchiosito.iisviolamarchesini.edu.it/j2/docs/15maggio/documento_15_maggio_2018_5H_meccanica.pdf).

The cultural impact of Leonardo da Vinci’s Lion is similar to contemporary AI discussions (Figure 14). Just as the Lion Robot captured the imagination of da Vinci’s contemporaries, AI continues to fascinate and provoke debate, raising questions about human creativity and the ethical implications of machines that can imitate it.

**Figure 15:**
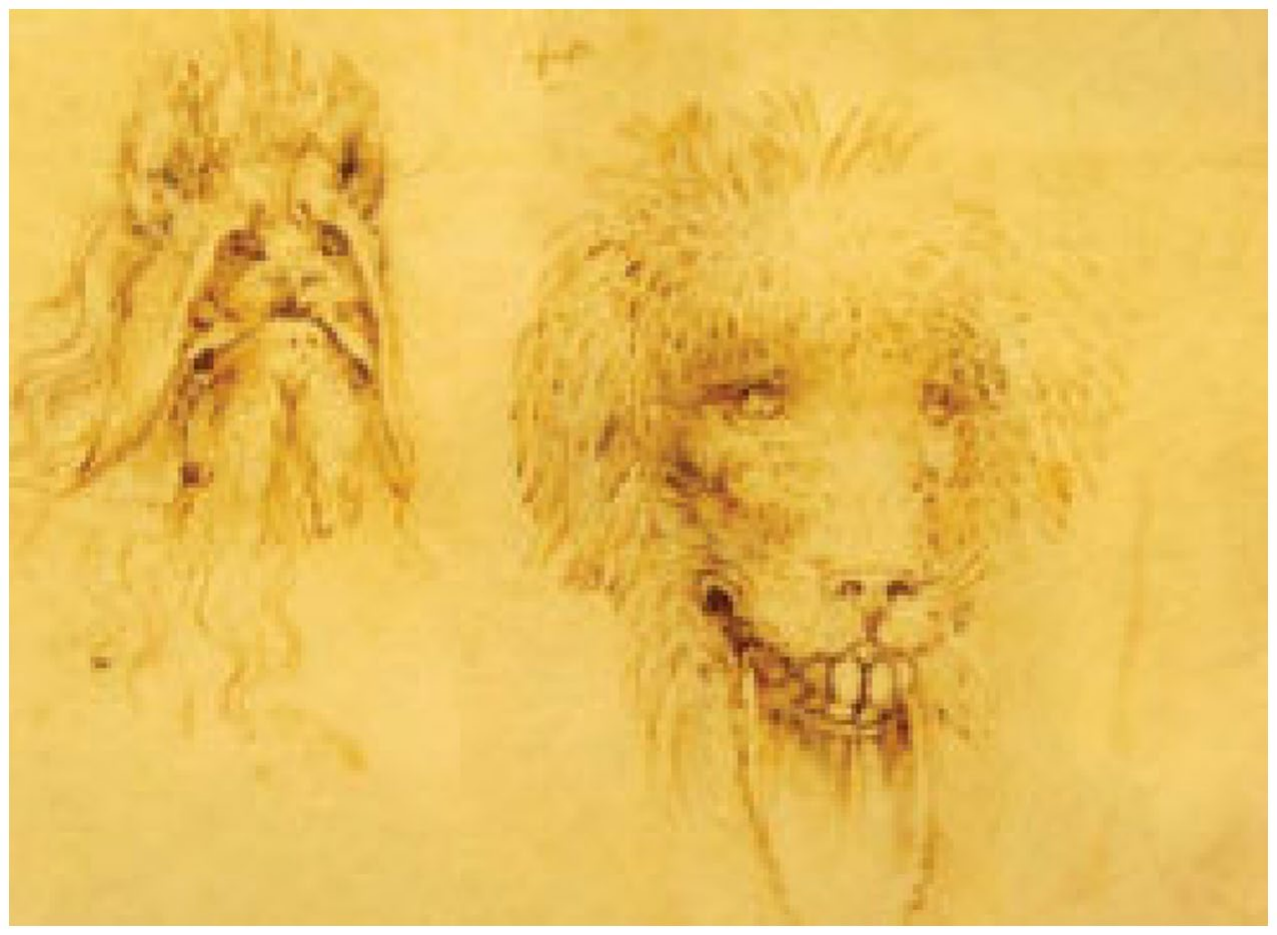
From Leonardo’s notebook: Codex Madrid 1 (found in Anderson, 2006) (https://www.edgeofyesterday.com/time-travelers/reconstruction-of-mechanical-lion).

The Lion Robot by Leonardo da Vinci (drawing can be seen in Figure 15) represents an early chapter in humanity’s never-ending quest to comprehend, replicate, and augment natural phenomena through technology. Despite being separated by centuries, the motivations behind da Vinci’s work and contemporary AI research are strikingly similar, reflecting the unchanging human desire to overcome biological limitations through innovation.

Leonardo da Vinci chose a lion as a model for his automaton because of the lion’s historical and cultural significance as a symbol of protection and strength. Throughout history, lions have been associated with protection and dominance. In the wild, they live in groups known as prides, led by dominant males who defend their territory and pride members. Similarly, Leonardo’s lion was created to move and present flowers, perhaps as a nod to the lion’s dual nature as a fierce predator and a member of a social, protective unit (Figure 15). The lion robot was a technical exercise and a manifestation of prevalent views on natural order and hierarchy, embodying the protective and commanding characteristics attributed to lions.

Analytically, Leonardo’s automata foreground the separation between appearance and agency. The mandate is symbolically rich but operationally fixed; control is embedded in mechanical sequencing rather than real-time oversight; and failure consists not in ethical misalignment but in the absence of adaptive judgement. The case therefore contributes to the comparative framework by illustrating how increasing representational sophistication does not entail increased moral or decision-making capacity.

## Comparative interpretation of ‘The Writer’ - Jaquet-Droz automata

**Figure 16:**
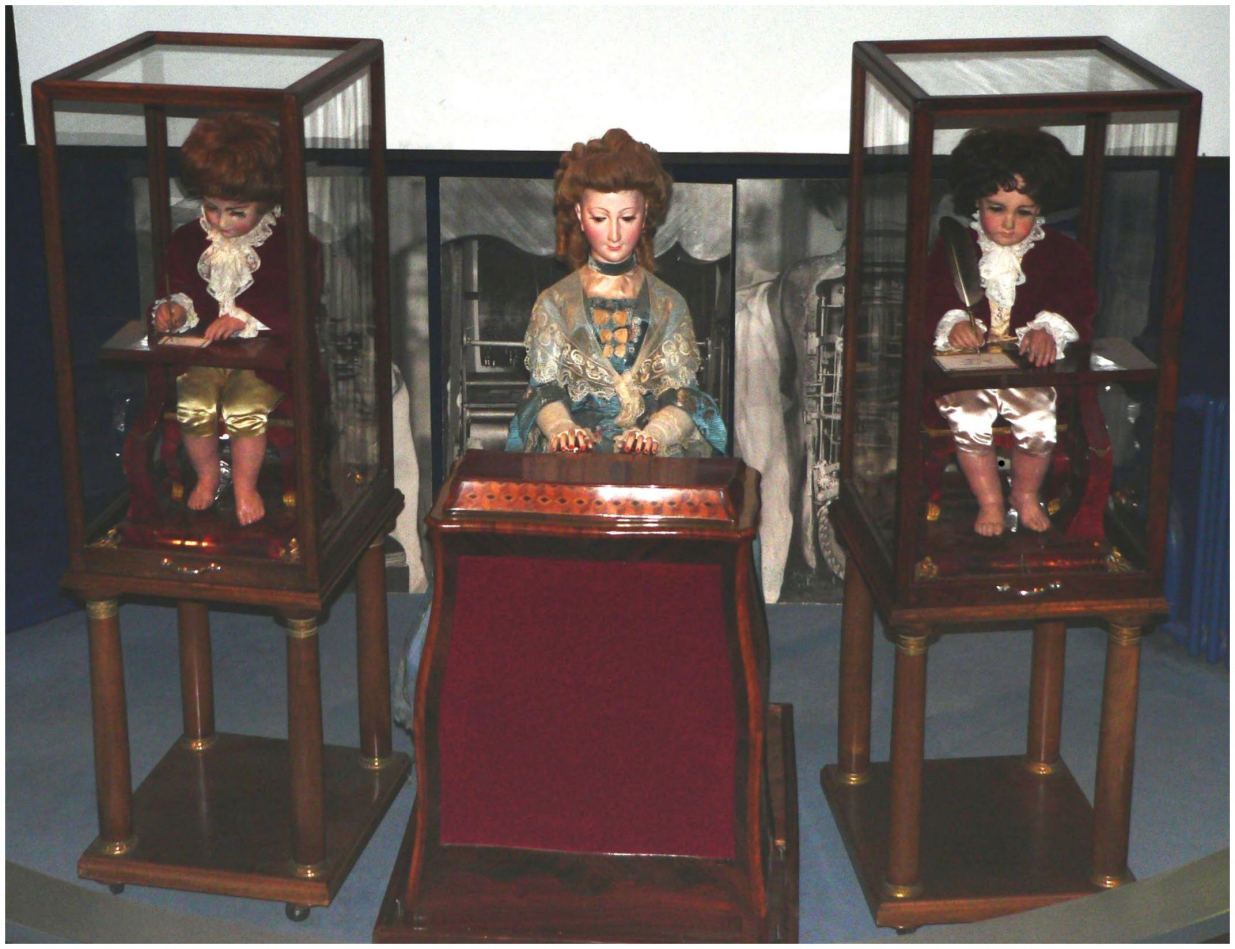
The Jaquet-Droz automata at the Musée d’Art et d’Histoire of Neuchâtel, in Switzerland (found in Yaghmour, 2019) (https://en.wikipedia.org/wiki/Jaquet-Droz_automata#/media/File:Automates-Jaquet-Droz-p1030472.jpg).

The Jaquet-Droz automata (Figure 16) are three doll automata that were created between 1768 and 1774 by Pierre Jaquet-Droz, his son Henri-Louis, and Jean-Frédéric Leschot. These automata include the musician, the draughtsman, and the writer dolls. They are all in working condition and are currently on display at the Musée d’Art et d’Histoire de Neuchatel in Switzerland. These dolls are the distant ancestors of modern-day computers. There was a fourth automaton known as ‘the Cave’, a large diorama consisting of a palace carved into a rock, gardens, and figurines. However, it has since vanished.

The ‘Writer’ automaton is a 70-centimetre-tall boy sitting on a stool at a mahogany table, as seen in Figure 16, found in Yaghmour (2019). Its mechanism comprises over 6,000 parts, making it the most complicated of the three previously mentioned automata. The automaton is programmable to write any 40-character text across three lines. It dips the quill into the inkwell with its right hand, turns its eyes toward the pen, shakes its head, and begins writing on a moving sheet of paper. After almost 250 years, it still works perfectly. It serves not only as a testament to the brilliance of Pierre Jaquet-Droz, but also demonstrates the capacity of machines to replicate aspects of human behavior without possessing underlying understanding. Observing the operation of this automaton, which many consider to be the ancestor of modern computers, reveals Jaquet-Droz’s genius.

The ethical dilemma encoded in the Writer automaton concerns programmability without understanding. The automaton executes symbolic instructions flawlessly yet lacks comprehension. This anticipates contemporary concerns regarding systems that generate linguistically coherent outputs without semantic awareness. The dilemma is not deception per se, but the delegation of symbolic authority to mechanisms incapable of contextual judgement.

From the perspective of the interpretive criteria, the Writer automaton isolates symbolic execution as a distinct form of delegated agency. Its mandate is precisely specified at the level of inscription, its control is preconfigured through mechanical programming, and its failure mode lies in the absence of semantic understanding despite syntactic correctness. This anticipates contemporary concerns regarding systems that produce coherent outputs without grounding or accountability.

## The emergence of thinking machines

**Figure 17:**
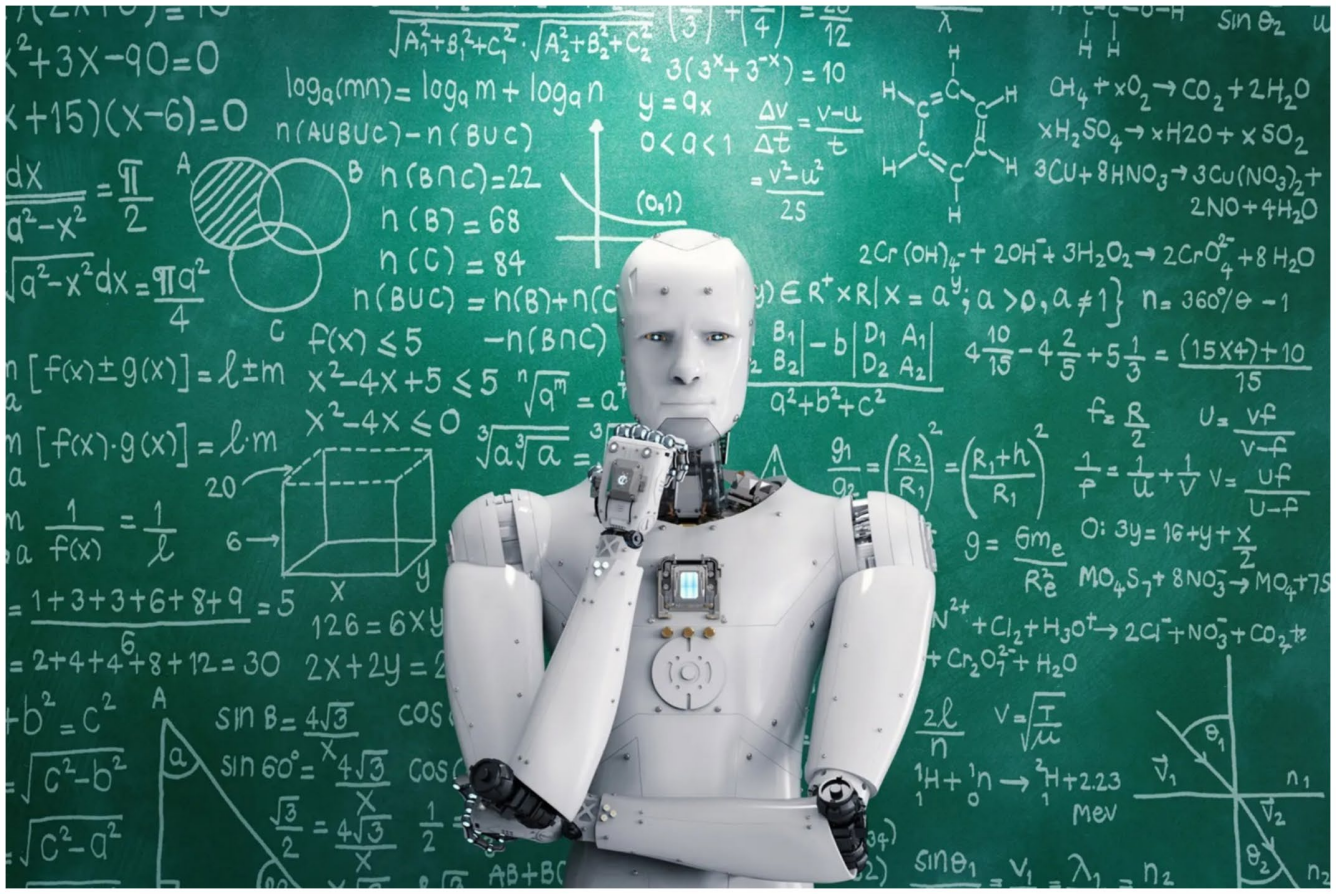
Thinking Machine as portrayed by Froelings (2018) (https://www.paymentsjournal.com/how-machine-learning-works-and-why-its-important/).

The story of Talos marks the first record of a long human obsession with robots that possess artificial intelligence, or in other words, thinking machines (Figure 17). A thinking machine (or intelligent machine) is a computer or robot with human intelligence (Beltzung, 2013). In the context of artificial intelligence, thinking machines represent AI systems capable of advanced reasoning, learning, and problem-solving. These machines can use various AI approaches, such as neural networks, deep learning, and reinforcement learning.

**Figure 18:**
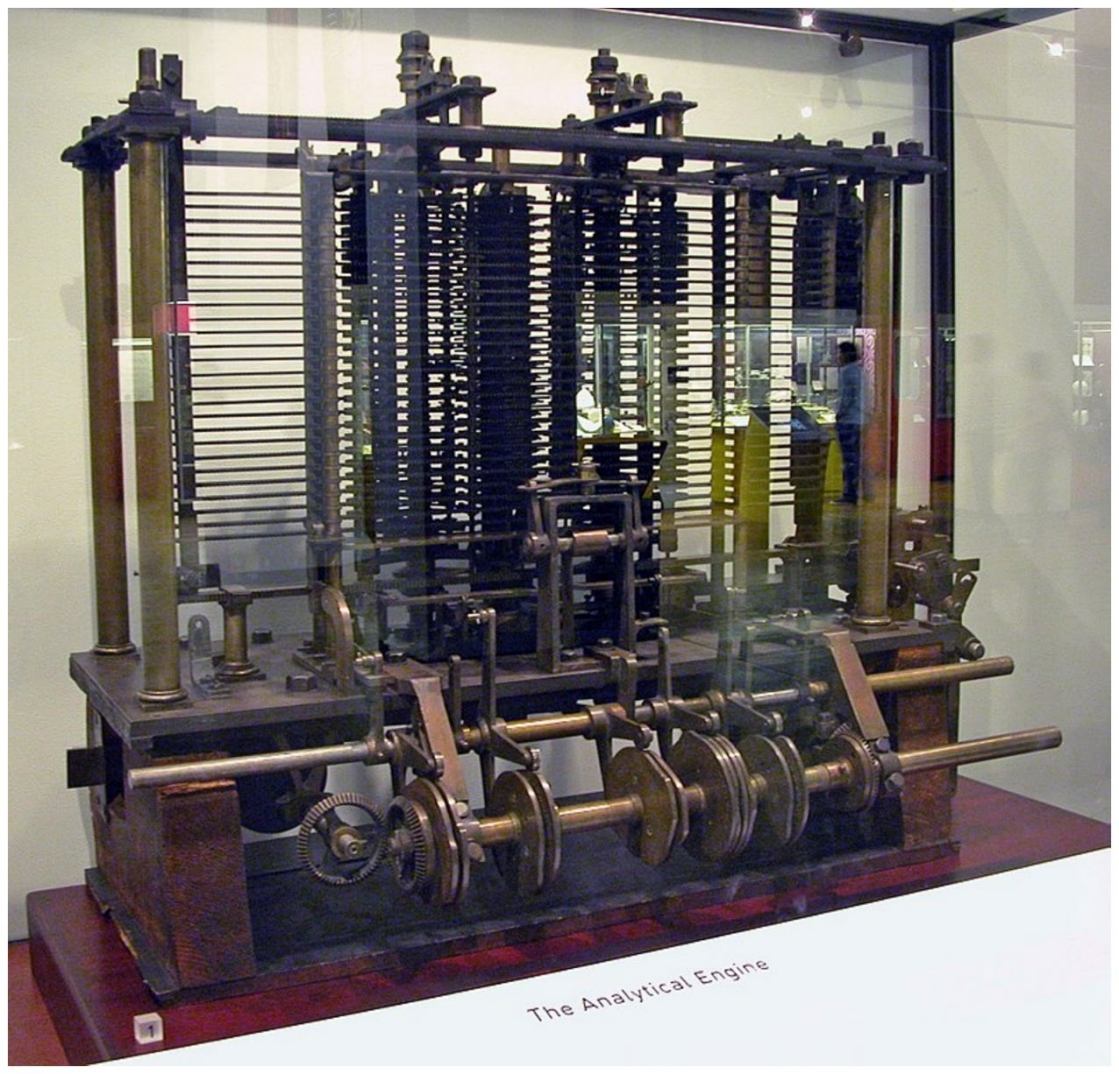
The Babbage analytical engine, 1833, is considered the first steam-powered computer. Babbage (1833) is considered by many to be the ‘Father of the Computer’ and his assistant, Lady Ada Lovelace, the ‘First Computer Programmer’ because she wrote mathematics problems for Babbage’s machines. © 2026 Bodleian Libraries, Science Museum Group (https://medium.com/tech-is-a-tool/building-the-modern-computer-babbages-analytical-engine-8179dedb08c8).

The British mathematician Charles Babbage developed the ‘Analytical Engine’ concept in the 1830s (Babbage, 1833). It was the first time anyone had thought about a machine that could think. Babbage was a well-respected mathematics professor at Cambridge University. However, he resigned from his position to dedicate all his time and efforts to his ground-breaking idea. Figure 18 shows a trial model of the Analytical Engine, taken in the Science Museum Group.

The Bodleian Library holds some records preserved from those days, including the Ada Lovelace and Charles Wheatstone paper published in Taylor’s Scientific Memoirs in August 1843.

**Figure 19:**
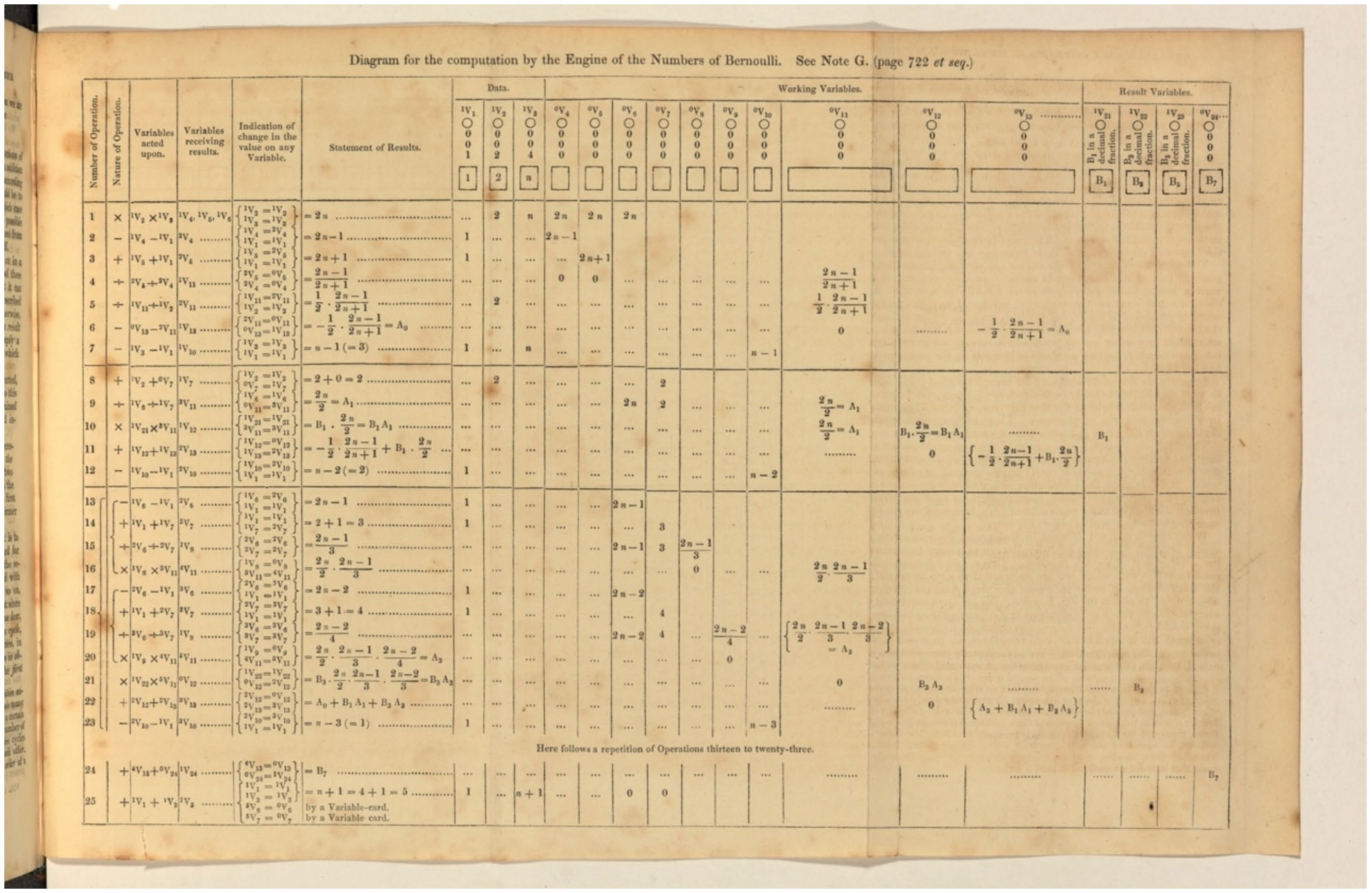
Note G © Magdalen College Libraries and Archives, Daubeny 90.A.11 (Lovelace, 1842) (https://blogs.bodleian.ox.ac.uk/adalovelace/2015/09/02/celebrate-ada-lovelace-in-oxford/).

The importance of Note G in the paper cannot be overstated (see Figure 19), as it effectively demonstrates the machine’s impressive capacity by calculating the ‘Bernoulli numbers’ (Hollings et al., 2020), which hold great significance in modern mathematics. These numbers are particularly well-suited for machine computation due to their recursive definition, allowing for calculating subsequent values based on previous ones. While there are multiple methods for computing these numbers, Lovelace opted not for the simplest option but to emphasize the engine’s capabilities. She noted that the goal was not simplicity or ease of computation but to showcase the machine’s impressive potential.

The cards provided instructions on how to compute the Bernoulli numbers. The paper explained in detail how to retrieve the various quantities needed from the Store, use them in the Mill, and move them back to the Store. To illustrate the process, a large table was used, with columns representing the values of the data, variables, and intermediate results at each step of the calculation performed by the engine.

This table is often referred to as the ‘first computer program.’ Still, Lovelace clarified that it ‘presents a complete simultaneous view of all the successive changes’ in the machine’s components as the calculation progresses (Lovelace, 1842). In modern computer science terms, the table represents an ‘execution trace.’ Had the concept existed then, the ‘programme’ would have been the deck of punched cards that caused the machine to make these successive modifications. Due to the need for more specific details in Babbage’s designs on how the cards would be manipulated, it is challenging to reconstruct the exact program. Geoff Toothill created a similar diagram one hundred years later, describing the operation of the ‘Manchester Baby,’ the first computer with a stored program, using a similar table to explain computation.

**Figure 20:**
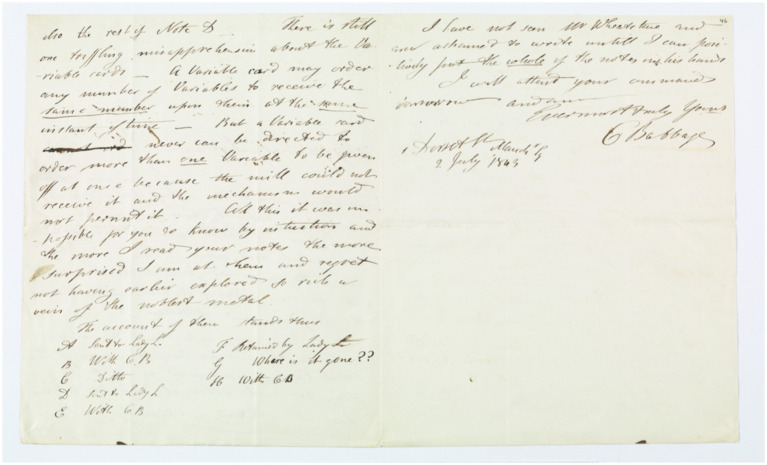
Charles Babbage’s letter to Ada Lovelace © Bodleian Library, University of Oxford (Hollings et al., 2018a) (https://blogs.bodleian.ox.ac.uk/adalovelace/2018/).

Lovelace and Babbage collaborated through exchanging letters and sharing versions of the table for the Bernoulli numbers (Figure 20). Their partnership reflects the common frustrations that all collaborators experience. Babbage expressed annoyance when they lost track of Note G, asking, ‘Where is it?’ The project ended with tempers flaring. Lovelace opposed Babbage’s addition of a strong critique of the British government in the paper, and Babbage declined her offer to become more involved in organizing the construction of the engine. However, Babbage continued to praise Lovelace, and wrote to Michael Faraday that Enchantress has cast a magical spell over the most abstract of sciences and grasped it with a force that few masculine intellects could have exerted over it.

Although Ada Lovelace and Charles Babbage never worked together again, they remained good friends. Lovelace used to write letters to Babbage and share with him information about the mathematics books she was reading her children’s growth, and the funny things her pets - dogs, chickens, and starlings - did. In the last year of Lovelace’s life, Babbage went with her to the Great Exhibition and suggested that she should wear worsted stockings, cork soles, and everything else that can keep her warm. Unfortunately, none of his machines were exhibited, disappointing him greatly (Hollings et al., 2018b).

These are just some of the many manuscripts held by the Bodleian Library on this topic.

### How is the work of Charles Babbage and Ada Lovelace related to the current generative AI?

The contributions of Charles Babbage and Ada Lovelace in the nineteenth century are considered foundational to computer science, including the contemporary field of generative AI. Babbage’s invention of the Analytical Engine, a mechanical general-purpose computer, paved the way for computational machinery. Ada Lovelace’s annotations to Babbage’s work demonstrated her understanding of the machine’s capabilities, which extended beyond simple calculations. Given the appropriate algorithms, Lovelace believed that the machine could manipulate symbols to create music and art. This vision prefigures the essence of generative artificial intelligence, which manipulates symbols (data) to produce novel, often creative outputs. Whether deep learning models generate text, images, or music, the underlying principle remains algorithmic manipulation of symbols to achieve a desired output. Lovelace’s concept of a machine capable of creative endeavors resonates strongly with the goals of generative AI, making her and Babbage intellectual forerunners of the field. Although the ethical and philosophical complexities we face today were largely unanticipated in their time, their contributions remain relevant and far-reaching.

Within the analytical framework, early computational systems mark a transition from mechanical to abstract delegation. The mandate becomes formalized in symbolic instructions, control is exercised through program design rather than physical intervention, and failure modes shift toward specification error rather than mechanical malfunction. This analysis considers early computational systems as a conceptual shift in how delegation is abstracted, highlighting how responsibility becomes increasingly mediated through symbolic specification rather than direct control.

## Graphical-textual examination grounded in an interpretive philosophical stance framing human narratives in the story of Pygmalion and Galatea

**Figure 21:**
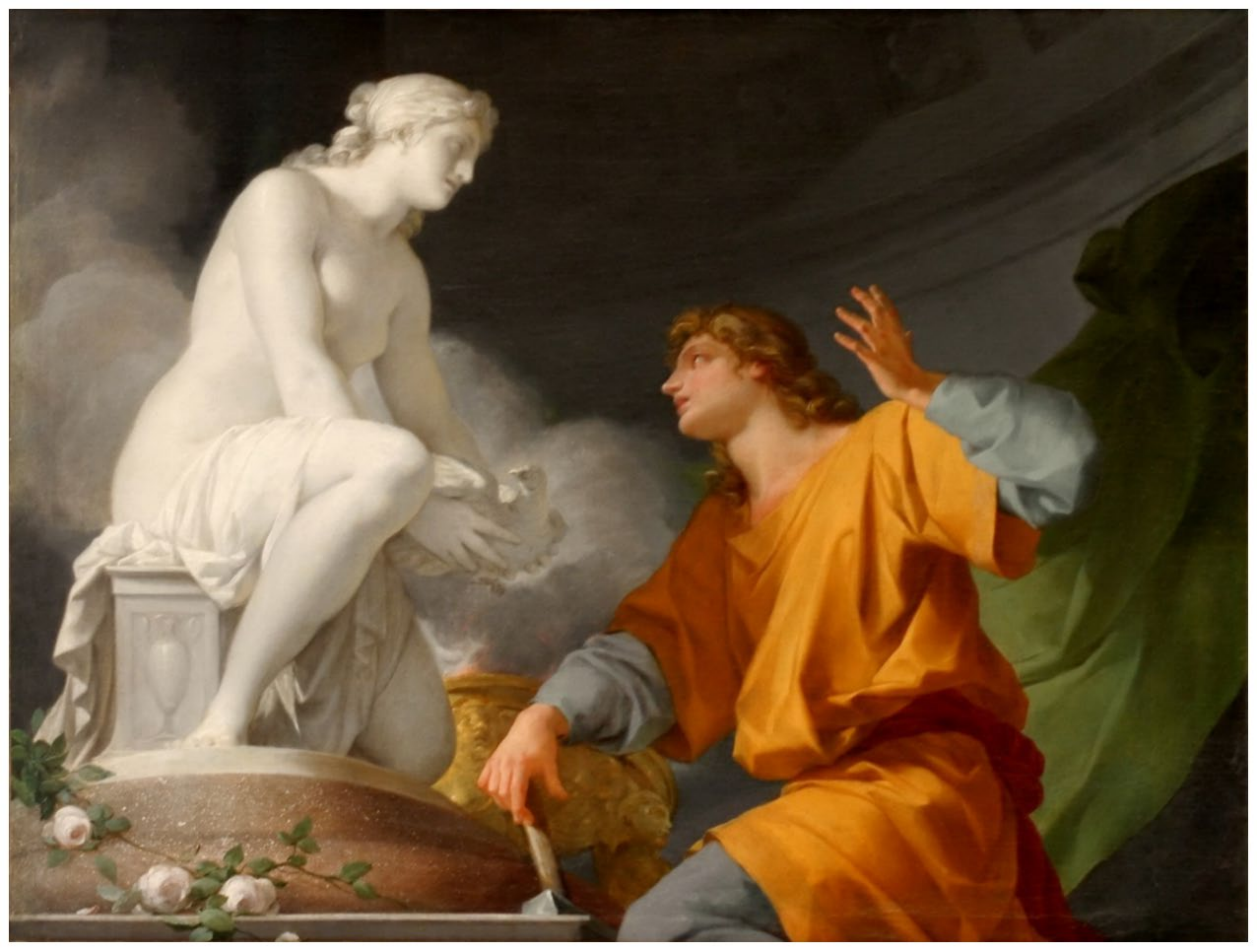
Pygmalion by Regnault (1786), Musée National du Château et des Trianons (https://commons.wikimedia.org/wiki/File:Ch%C3%A2teau_de_Versailles,_salon_des_nobles,_Pygmalion_priant_V%C3%A9nus_d%27animer_sa_statue,_Jean-Baptiste_Regnault.jpg).

The analysis adopts an interpretive philosophical stance that examines how cultural narratives represent artificial agency, while maintaining a normative commitment that moral responsibility remains grounded in human agents. The best story to end this article is the story of ‘Pygmalion and Galatea’ (Hamilton, 1953). In Greek mythology, the king and sculptor Pygmalion was a legendary figure from Cyprus. Pygmalion is best known from Ovid’s narrative poem Metamorphoses, in which a sculptor falls in love with a statue he carved, see Figure 21 (Regnault, 1786). The image in Figure 21, is the representation of Pygmalion by Regnault (1786), and can be found in the National Museum of the Château and the Trianons. The primary source for this narrative is Ovid’s Metamorphoses (Ovid, 2004), which provides the canonical account of the transformation.

**Figure 22:**
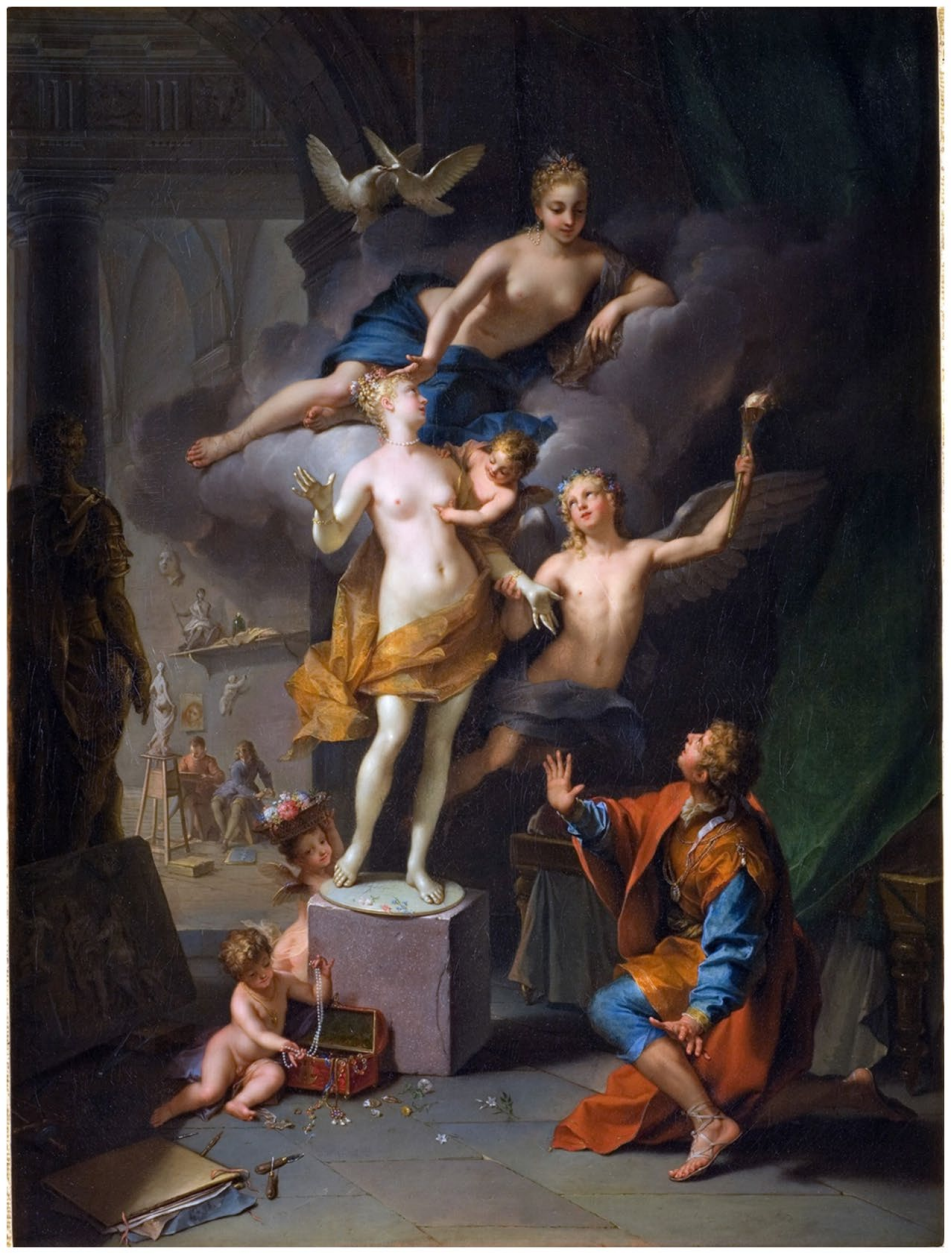
Pygmalion Adoring His Statue by Raoux (1717) (https://commons.wikimedia.org/wiki/File:Pygmalion_(Raoux).jpg).

In book 10 of Ovid’s Metamorphoses, Pygmalion is a Cypriot sculptor who carved a woman out of ivory. Post-classical sources name her Galatea. According to Ovid’s account, Pygmalion became disgusted with the behavior of the Propoetides of Cyprus who were involved in prostitution and started to despise the imperfections that nature had bestowed upon women. As a result, he decided to remain unmarried and took up sculpture as a hobby. He created a sculpture of a woman that was so beautifully crafted that he fell in love with it. Pygmalion caressed and fondled the sculpture, brought it gifts, and even made a luxurious bed for it (Figure 22; Raoux, 1717).

**Figure 23:**
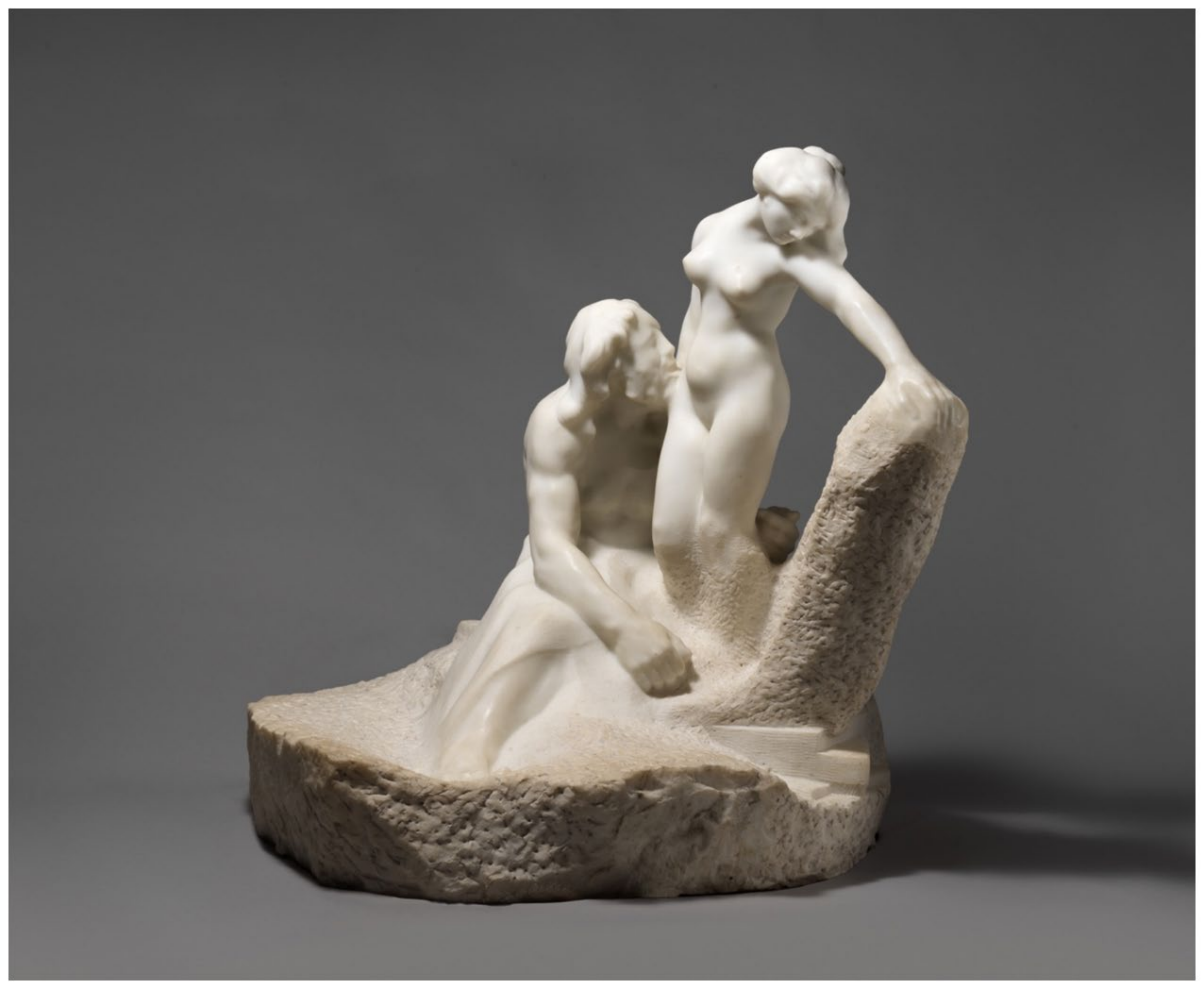
Pygmalion and Galatea by Rodin (1889) (https://www.metmuseum.org/art/collection/search/191292, https://commons.wikimedia.org/wiki/File:Pygmalion_and_Galatea_MET_7436.jpg).

Pygmalion awaited Aphrodite’s festival day eagerly and made offerings at her altar. He secretly wished for a bride who resembled his ivory statue (Figure 23), but he was afraid to admit it. When he returned home and kissed the statue, he discovered its lips were warm. After kissing it again, he realized the ivory had lost its hardness. Aphrodite granted Pygmalion’s wish, and the ivory sculpture transformed into a woman. They got married, and according to Ovid, they had a daughter named Paphos. However, other accounts suggest that Paphos was a son, and their daughter was Metharme. Ovid’s mention of Paphos suggests that he had access to more detailed information than the source for a passing mention of Pygmalion in Pseudo-Apollodorus’ Bibliotheke, a Hellenic mythography of the 2nd-century AD. It is possible that he drew on Clement of Alexandria’s paraphrase of a lost narrative by Philostephanus.

The Figure 23 is the final image in this analysis, and is used to reinforce the conclusions that humans have long desired for artificial connection. The statue shown in Figure 23 (Raoux, 1717), clearly shows that that the artificial creations human create, are result of the human desires, needs, fears, and these creations serve to fulfil some missing human needs. If future artificial creations lead to the demise of humanity, this event would be reflective of the values and the desires of the humans that created such artificial creations. However, as we have determined in this article, the desires for security, connection, love, protection, is not developed by artificial intelligence. This is merely a result of the human requirement for something that we fear, or something that we desire. Hence, artificial intelligence is simply a mirror of humanity. If artificial intelligence leads to our demise, such outcomes would reflect the values, design choices, and governance structures embedded by human actors rather than independent properties of artificial systems.

### Artificial animation and the threshold of moral agency

The Pygmalion narrative introduces an important complication absent in Talos and the Golem. Once animated by Aphrodite, Galatea is no longer a bounded artifact; she becomes a living human being within the mythological ontology. The ethical structure therefore shifts. Galatea, once alive, is capable of reciprocity, relationship, and participation in the moral community. To be more specific, the Pygmalion narrative is analytically distinct from the preceding cases because it operates across two structurally different stages: projection onto an artifact and the subsequent transformation into a living subject. The first stage is relevant to delegated artificial agency and the risks of anthropomorphism; the second introduces a separate ethical question concerning the creation of morally considerable beings. The analysis that follows treats these stages separately to avoid conflating projection with genuine moral agency.

This transformation marks a threshold distinction. Prior to animation, the statue functions as an object of projection. After divine intervention, it becomes a subject. The moral question is no longer about delegation, but about the ethics of creation and recognition. This distinction clarifies the contemporary debate. Current AI systems do not cross this threshold. They simulate intention and emotion but lack consciousness, sentience, or reciprocal moral standing. The ethical risks they pose arise from projection and misattribution rather than from genuine moral agency.

The Galatea narrative therefore does not undermine the argument that artificial agents historically lacked moral status. Instead, it isolates the conceptual boundary at which moral agency would become relevant. The modern anxiety surrounding advanced AI is precisely anxiety about whether such a threshold could ever be crossed. The present paper argues that contemporary systems remain structurally within the domain of delegated agency, not independent moral subjecthood.

After all, humans have desired weaponising artificial intelligence since the Ancient Greece, as we can see in the silver didrachma from Phaistos, Crete (in Figure 5), and that is long before humans had the capability to build any form of artificial intelligence, even the most basic forms of artificial intelligence were developed long after the Ancient Greeks developed the idea of Talos (Robertson, 1977).

### How does the story of Pygmalion and Galatea relate to the human interactions with AI?

The story of Pygmalion and Galatea draws remarkable parallels with the current state of generative AI. It tells the story of a sculptor who falls in love with a statue he created, which then comes to life. Similarly, algorithms used by generative AI have evolved to interpret and generate intricate patterns in various domains, such as natural language processing, image synthesis, and more. The advanced AI models can mimic human-like characteristics and responses, blurring the line between human-generated and AI-generated content. However, unlike Galatea, even the most advanced AI lacks genuine consciousness and emotional depth. The ethical implications of humanizing AI, including gender and racial bias, are subjects of ongoing academic scrutiny. Thus, the story of Pygmalion serves as both a metaphor for technological ambitions and a cautionary tale about the complexities of imbuing inanimate objects with ‘life.’

The concept of endowing artificial intelligence with human-like traits, including emotions, love, relationships, reproduction, and coexistence, is fascinating but also a challenging prospect. AI ethics and philosophy have already explored the idea of AI possessing consciousness or emotional intelligence. In the future, AI models may be capable of simulating emotional states, engaging in meaningful relationships, and becoming companions or caregivers in social networks, healthcare, and even families. AI could collaborate with humans in generative tasks like digital art creation or scientific research, which could be considered a form of procreation that transcends biology. However, integrating advanced AI into society raises ethical dilemmas such as the rights of AI entities, the risk of emotional manipulation, and the potential to exacerbate social disparities. Therefore, while incorporating human-like traits in AI is an intriguing topic, it requires careful ethical and philosophical examination to ensure that technological advancement aligns with human values.

## Discussion

This Discussion synthesizes the historical analyses and normative argument advanced in the paper by foregrounding the Normative Model of Delegated Moral Agency in AI (Figure 3). The figure is introduced here to consolidate the paper’s central finding: that artificial intelligence systems, despite increasing technical sophistication, do not constitute autonomous moral agents but operate as sites of delegated human authority. The model provides a conceptual bridge between the historical cases examined earlier and contemporary AI ethics debates, enabling a precise account of where moral responsibility resides and how it is frequently obscured.

### Normative position and ontological commitment

While the historical analysis examines how cultures have represented artificial agency, the normative argument advanced in this paper is not merely descriptive. The claim that moral responsibility remains human is a philosophical position grounded in standard accounts of moral agency requiring intentionality, consciousness, and normative understanding.

The study therefore distinguishes between interpretive analysis of cultural representations and a normative commitment regarding agency. Artificial systems may be socially perceived as agents; this does not entail that they satisfy the conditions for moral subjecthood. Clarifying this distinction resolves any tension between interpretive method and normative conclusion.

Figure 3 visualizes this argument by anchoring moral responsibility in human moral agency, defined through intentions, values, and governance decisions, while positioning artificial agents, mythic, mechanical, and computational, as entities whose agency is derivative rather than intrinsic. The inward-pointing control and accountability mechanisms depicted in the figure emphasize that oversight, intervention, and responsibility must flow from human institutions toward artificial systems. The critical claim placed outside the model, that ethical failure occurs when responsibility is displaced from humans to artifacts, captures the normative core of the study and explains why responsibility gaps persist even in technically well-aligned systems.

A central clarification required for the governance thesis advanced in this paper concerns the distinction between the *moral location* of responsibility and the *practical enforceability* of that responsibility. These dimensions are analytically separable and must not be conflated.

The claim that moral responsibility remains irreducibly human is a normative thesis concerning attribution: artificial systems, regardless of autonomy or capability, do not constitute bearers of moral obligation. Responsibility attaches to designers, deployers, operators, institutional leaders, and public authorities. This attributional claim holds even in scenarios involving catastrophic or existential risk. The fact that a system may act unpredictably does not alter the moral ontology of responsibility.

However, recognizing the location of responsibility does not resolve the separate question of enforceability. Enforcement depends upon institutional capacity, jurisdictional reach, geopolitical stability, and incentive compatibility. Under contemporary conditions of technological acceleration and multipolar competition, enforcement is non-trivial. Diffusion of capability through open-source models, cross-border compute infrastructure, and private-sector concentration of technical expertise complicate traditional regulatory approaches. The governance thesis therefore does not deny feasibility constraints; rather, it asserts that these constraints are institutional and geopolitical problems rather than evidence of machine moral agency.

Distinguishing these two dimensions prevents a common misunderstanding: acknowledging governance failure does not imply that governance is simple. It implies that the locus of intervention remains institutional even when enforcement is difficult. Alignment, on this account, is not reducible to algorithmic optimization; it is a coordination problem embedded in legal systems, corporate structures, and international order.

If AI risk is understood as a governance failure, then structural impediments to governance must be addressed explicitly. Four constraints are particularly salient under current geopolitical conditions.

- Geopolitical multipolarity: Advanced AI development is distributed across major powers with divergent political systems and strategic objectives. Regulatory convergence is therefore contingent upon fragile diplomatic alignment. In a security-dilemma context, unilateral restraint may be perceived as strategic disadvantage.
- Competitive acceleration dynamics: Corporate and national actors operate under innovation incentives that reward capability scaling. Competitive pressures can produce race dynamics in which safety investment is subordinated to market or strategic positioning.
- Open-source diffusion: The proliferation of openly released model weights reduces centralized control over deployment pathways. While open science has epistemic benefits, it complicates enforcement of safety standards and compute restrictions.
- Jurisdictional asymmetry: Enforcement capacity varies dramatically across regulatory environments. Firms may arbitrage governance by relocating development or infrastructure.

Recognizing these constraints strengthens rather than weakens the governance thesis. It demonstrates that alignment problems are embedded in collective action structures analogous to climate governance and nuclear non-proliferation. Technical alignment research remains necessary, but absent institutional coordination, even well-aligned systems may be deployed in destabilizing ways.

Governance must be analyzed across multiple levels rather than presupposed as a monolithic authority.

- Corporate governance concerns internal risk management, board oversight, model evaluation protocols, red-teaming integration, and compute governance.
- National regulatory governance includes licensing regimes, safety audits, liability allocation, procurement constraints, and compute reporting requirements.
- International coordination mechanisms encompass export controls, shared safety standards, incident reporting frameworks, and treaty-level constraints on high-risk capabilities.
- Technical architecture governance embeds constraints directly within systems, including sandboxing, capability caps, model evaluations prior to scaling, and structured deployment gating.

Distributing responsibility across these layers avoids unrealistic centralisation while preserving the normative claim that responsibility remains human. Alignment becomes a cross-layer coordination task rather than a purely algorithmic objective.

Beyond its illustrative role, the model in Figure 3 enables several substantive findings that extend current AI ethics discourse. First, the analysis demonstrates that alignment problems are governance problems before they are technical ones. This argument does not deny the epistemic difficulty of technical alignment research; rather, it maintains that institutional specification of goals precedes and constrains algorithmic optimization. Across the historical cases, artificial agents fail not because their operational logic is unclear, but because the human objectives they embody are under-specified, conflicting, or insufficiently constrained. Talos’ catastrophic vulnerability, the Golem’s conditional obedience, and the rigidity of early automata all reveal that misalignment originates in human design decisions rather than in emergent machine behavior. This finding challenges contemporary tendencies to treat alignment as a predominantly algorithmic challenge, instead reframing it as a question of institutional clarity and normative intent.

Second, the study shows that responsibility gaps are best understood as emerging from how delegation is structured across distributed socio-technical systems, not by complexity. Figure 3 clarifies that when artificial agents act at scale or speed, responsibility becomes diffused unless explicit mechanisms preserve traceability to human decision-makers. The historical record illustrates that societies have long struggled with this problem: divine proxies, legal persons, and mechanical agents have all been used to act on behalf of humans while simultaneously distancing those humans from the consequences of action. Modern AI systems intensify this pattern, but they do not introduce it. The novelty lies in scale, not in ethical structure.

This position departs from the dominant account in contemporary AI ethics, which explains responsibility gaps primarily in terms of opacity, epistemic limitation, and diminished human control. On this view, the complexity of machine learning systems, particularly those based on high-dimensional statistical models, prevents any individual human actor from possessing sufficient understanding or control to ground responsibility attribution. Responsibility gaps therefore arise because no agent satisfies the epistemic and control conditions typically required for moral accountability.

The present argument does not reject this diagnosis but reinterprets it. Opacity and limited control are not independent sources of responsibility gaps; they are consequences of how authority has been delegated across distributed socio-technical systems. When design, training, deployment, and governance are fragmented across multiple actors without clear accountability structures, epistemic limitations become institutionally embedded rather than merely technically imposed. Responsibility gaps therefore persist not because systems are inherently inscrutable, but because delegation is not accompanied by mechanisms that preserve traceability, oversight, and enforceable responsibility across the lifecycle.

This reframing shifts the analytical focus from the intrinsic properties of machine learning systems to the governance structures within which they operate. Complexity and opacity intensify the problem, but they do not fundamentally alter its structure: responsibility remains attributable to human actors, even when it is rendered difficult to operationalize in practice.

The claim that AI introduces problems of scale rather than structure requires clarification. Contemporary systems operate at speeds that exceed human reaction time. In domains such as autonomous driving, collision avoidance, or high-frequency trading, human-in-the-moment oversight is infeasible. However, the absence of real-time intervention does not relocate moral agency to the system. It shifts responsibility temporally rather than ontologically. Oversight occurs ex ante (through design constraints, training objectives, simulation environments, and certification) and ex post (through liability regimes and forensic audit), even if not synchronously.

The ethical structure therefore remains one of delegation: humans predefine optimization criteria, safety thresholds, and deployment contexts. The split-second decision of an autonomous vehicle reflects encoded human trade-offs rather than spontaneous machine morality. What changes is the compression of execution time, not the locus of responsibility. Scale and speed intensify risk by magnifying the consequences of design error. They do not transform artificial systems into moral agents. The governance challenge is to ensure that ex ante specification and ex post accountability compensate for the impossibility of *in situ* human deliberation.

The governance model developed in this paper can be illustrated through contemporary large language model (LLM) deployment. LLMs exhibit artificial agency by generating context-sensitive outputs under probabilistic optimization, yet their authority is entirely delegated through human design decisions: training corpus construction, objective function specification, reinforcement learning protocols, deployment interfaces, and content moderation policies.

Responsibility gaps arise when harmful outputs are attributed to “the model” rather than to upstream decisions regarding data selection, reward shaping, and deployment governance. Applying the criteria of proper delegation—auditability, interruptibility, capability bounding, and compute oversight—clarifies institutional obligations. For example, post-deployment monitoring, structured red-teaming, and staged capability release function as contemporary analogs of the override mechanisms identified in the Golem narrative.

This application demonstrates that the framework developed here is not merely historical or conceptual. It provides operational guidance for current AI governance by specifying how responsibility can remain traceable even when artificial systems exhibit high degrees of behavioral autonomy.

Third, the findings highlight the central importance of override mechanisms and interruptibility as ethical safeguards rather than merely technical features. The Golem’s deactivation ritual and Talos’ single-point-of-failure anticipate contemporary kill-switch and human-in-the-loop requirements. Figure 3 situates these mechanisms within a normative framework, showing that their ethical function is to reaffirm human moral authority rather than simply to prevent system malfunction. This reframing has direct implications for AI governance: interruptibility should be understood as a moral requirement tied to accountability, not merely as a safety engineering feature.

Fourth, the study identifies anthropomorphism as a persistent ethical risk rather than a superficial cultural phenomenon. The Pygmalion narrative demonstrates how emotional projection onto artificial entities encourages the misattribution of moral agency. In contemporary contexts, conversational and generative AI systems amplify this tendency by simulating empathy, intention, and creativity. Figure 3 counters this risk by visually decoupling apparent agency from moral responsibility, reinforcing the argument that attributing agency to machines undermines ethical scrutiny of human actors and institutions.

Taken together, these findings support a broader normative conclusion: AI ethics failures recur when human responsibility is rendered opaque by delegation, scale, or narrative framing. The contribution of this study lies not in proposing new ethical principles, but in clarifying the conditions under which existing principles repeatedly fail to be operationalized. By integrating historical analysis with a normative conceptual model, the paper shows that ethical governance of AI requires sustained attention to human agency, institutional design, and accountability structures, rather than an exclusive focus on technical autonomy.

The normative conclusions drawn in this study do not rest on superficial analogy between myth and machine. Rather, each historical case isolates a structural feature of delegated agency: bounded optimization (Talos), conditional activation and override (Golem), projection and anthropomorphism (Pygmalion), and programmability (Lovelace and Babbage).

The inference to contemporary AI governance proceeds by structural comparison rather than narrative similarity. Where artificial systems execute pre-specified optimization under constrained objectives, risks arise from mis-specified goals, brittle generalization, and insufficient oversight. These risks are homologous to those observed in earlier artifacts, even if the technological substrate differs.

The argument therefore does not claim that myth predicts machine learning. It claims that human delegation of authority to non-deliberative systems generates recurrent governance problems. The contemporary novelty lies in magnitude and integration, not in moral architecture.

In this sense, artificial intelligence does not represent a rupture in moral history but a continuation of long-standing human practices of creating powerful intermediaries. The ethical challenge, as Figure 3 makes explicit, is not to constrain machines as moral actors, but to prevent humans from relinquishing moral responsibility to the artifacts they create.

The concept of “improper delegation” requires technical specification if it is to guide contemporary AI governance. Proper delegation in frontier systems can be operationalized through five enforceable criteria:

- Auditability: Model development, training data provenance, and deployment pathways must be traceable and independently reviewable. Logging and documentation standards should enable post-incident attribution.
- Interruptibility: Systems must remain technically and institutionally interruptible. Kill-switch mechanisms, deployment gating, and compute access controls are ethical requirements, not optional safety features.
- Reversibility: High-risk capability releases should be staged and reversible where feasible, preventing irreversible diffusion of dangerous functionality.
- Capability bounding: Explicit constraints on model scaling, autonomy, and integration into critical infrastructure reduce unbounded delegation.
- Compute monitoring and reporting: Frontier training runs should be subject to reporting thresholds and oversight mechanisms proportional to risk.

Improper delegation occurs when artificial systems are granted decision authority without these constraints, producing responsibility diffusion and enforcement failure. Proper delegation preserves human moral authority while enabling controlled technological innovation.

Figure 4 formalizes how moral agency and responsibility remain anchored in human actors despite the delegation of decision-making to artificial agents. Artificial agents, mythic, mechanical, and computational, operate with delegated agency, while responsibility remains with humans. Control and accountability mechanisms (human-in-the-loop oversight, design constraints, override or kill switches, and institutional accountability) enforce human values, enable intervention, and preserve governance. Ethical failure occurs when responsibility is displaced from human moral agency to artificial systems, producing responsibility gaps and loss of effective oversight.

## Conclusion

This study has advanced a normative and historically grounded account of artificial intelligence ethics by demonstrating that contemporary concerns regarding AI autonomy, alignment, and risk are not unprecedented technical phenomena but recurring consequences of how humans delegate moral authority to artificial agents. By analyzing mythological, mechanical, and early computational artifacts as conceptual models of artificial agency, the paper has shown that the ethical challenges associated with AI consistently originate in human intentions, design choices, and governance structures rather than in machine autonomy itself.

The primary contribution of the study lies in its reframing of AI ethics from a technology-centric problem to a governance-centric one. Across the cases examined—Talos, the Golem, automata, and early programmable machines—artificial agents exhibit bounded autonomy, operational vulnerability, and dependence on human oversight. These structural features closely correspond to contemporary AI systems, indicating that responsibility gaps and alignment failures emerge when moral responsibility is externalized to artifacts without sufficient mechanisms for control, override, and accountability. The normative model of delegated moral agency developed in this paper makes explicit that artificial systems act as intermediaries of human authority and that ethical failure occurs when this delegation obscures human responsibility.

By integrating historical analysis with a conceptual ethical framework, the study clarifies why technical solutions alone cannot resolve AI ethics challenges. Improved alignment, robustness, or optimization does not address the underlying problem if institutional arrangements fail to preserve traceable accountability and meaningful human oversight. The analysis further identifies anthropomorphism and affective projection as persistent risks that encourage the misattribution of moral agency to machines, thereby weakening ethical scrutiny of the human actors and organizations responsible for their deployment.

The implications of these findings are direct for AI governance and policy. Effective ethical governance requires explicit recognition that moral agency remains human, alongside institutional mechanisms that enforce responsibility, enable intervention, and prevent moral displacement. Rather than treating AI as an autonomous moral actor, governance frameworks must focus on design accountability, decision provenance, and enforceable oversight across the AI lifecycle.

In conclusion, artificial intelligence does not represent a rupture in moral history but a continuation of long-standing human practices of creating powerful intermediaries. The ethical challenge is not to constrain machines as moral agents, but to ensure that humans do not abdicate responsibility to the artifacts they design and deploy. Recognizing AI as a mirror of human moral agency provides a more precise foundation for responsible innovation and for the development of governance structures capable of addressing the real sources of ethical risk. This argument does not deny that contemporary AI systems introduce genuine challenges related to opacity, epistemic limitation, and diminished human control. Rather, it situates these challenges within the broader structure of delegated authority, showing that they arise from how responsibility is distributed across socio-technical systems rather than from a transfer of moral agency to machines. Addressing responsibility gaps therefore requires not only technical transparency, but institutional mechanisms that preserve attribution, oversight, and accountability.

## Data Availability

The original contributions presented in the study are included in the article/supplementary material, further inquiries can be directed to the corresponding author.
